# Wildfire Smoke Exposure and Cause-Specific Hospitalization in Older Adults

**DOI:** 10.1001/jamanetworkopen.2025.7956

**Published:** 2025-04-30

**Authors:** Sofia L. Vega, Marissa L. Childs, Sarika Aggarwal, Rachel C. Nethery

**Affiliations:** 1 Department of Biostatistics, Harvard T.H. Chan School of Public Health, Boston, Massachusetts; 2 Center for the Environment, Harvard University, Boston, Massachusetts

## Abstract

**Importance:**

The escalating intensity of wildfires in the western US is increasing exposure to smoke pollution. Previous studies of wildfire smoke and health have primarily focused on mortality and respiratory and cardiovascular events, with limited research on broader health impacts or on the shape of concentration-response curves.

**Objective:**

To characterize the associations between exposure to smoke-specific fine particulate matter (PM_2_*_._*_5_) and cause-specific hospitalizations among older adults in the western US.

**Design, Setting, and Participants:**

This retrospective cohort study used Medicare inpatient claims data from 2006 to 2016 linked with machine learning–derived smoke-specific PM_2_*_._*_5_ to assess associations between smoke PM_2_*_._*_5_ and hospitalization rates. Participants included Medicare beneficiaries aged 65 years or older who lived in a western US state (ie, Arizona, California, Colorado, Idaho, Montana, Nevada, New Mexico, Oregon, Utah, Washington, or Wyoming). Analyses were conducted from October 2023 to February 2025.

**Exposures:**

Daily county-level smoke-specific PM_2_*_._*_5_ concentrations were estimated from machine learning models trained on monitor and satellite data.

**Main Outcomes and Measures:**

Daily county-level rates of unscheduled hospitalization for each of 13 broad cause categories. To characterize the association between each cause of hospitalization and smoke PM_2.5_, distributed lag models were fitted with hospitalization rates modeled as a function of same-day smoke PM_2.5_ exposure and exposures on each day of the preceding week, using splines on exposure to allow for nonlinearity.

**Results:**

The study included 10 369 361 individuals (mean [SD] age, 74.7 [7.9] years; 4 862 826 male [46.9%]; 5 506 535 female [53.1%]; 373 252 Black [3.6%]; 420 577 Hispanic [4.1%]; and 8 365 607 White [80.7%]), 57 million person-months of follow-up, and 4.7 million unscheduled hospitalizations. Smoke PM_2_*_._*_5_ concentration-response curves for respiratory hospitalizations and cardiovascular hospitalizations were flat at lower concentrations but showed increasing trends at concentrations above 25 μg/m^3^. On average, daily hospitalizations (per 100 000) increased by 2.40 (95% CI, 0.17 to 4.63) for respiratory concerns when increasing same-day and preceding week smoke PM_2.5_ concentrations from 0 to 40 μg/m^3^; hospitalizations for cardiovascular concerns increased by 2.61 (95% CI, –0.09 to 5.30), a difference that was not statistically significant. No associations were observed for other causes of hospitalization.

**Conclusions and Relevance:**

In this cohort study, exposure to high levels of smoke pollution was associated with an increase in hospitalizations for respiratory diseases. These findings underscore the need for interventions to mitigate the health impacts of wildfire smoke exposure.

## Key Points

**Question:**

Is short-term exposure to air pollution from wildfire smoke associated with cause-specific hospitalizations among older adults in the western US from 2006 to 2016?

**Findings:**

In this cohort study of 10 369 361 Medicare beneficiaries, smoke pollution exposure was nonlinearly associated with respiratory hospitalization rates, with elevated rates occurring at higher concentrations. Increases for cardiovascular disease hospitalizations were not statistically significant, and no associations were observed for other causes of hospitalization.

**Meaning:**

These findings suggest that exposure to high concentrations of smoke pollution may be associated with respiratory, and possibly cardiovascular, health risks in older adults, highlighting the need for interventions to reduce exposures.

## Teaser

This cohort study examines the association of exposure to smoke fine particulate matter and cause-specific hospitalizations among adults aged 65 years and older residing in the western US during wildfire season from 2006 to 2016.

## Introduction

In recent years, the western US has faced devastating consequences as wildfires have surged in frequency and intensity.^1^ Due to the influence of climate change, continued escalation of wildfires in the western US is expected throughout the 21st century.^2^ In addition to the destruction brought directly by wildfires themselves, their smoke plumes can extend for thousands of miles, adversely affecting the well-being of far-flung communities. Indeed, wildfire smoke is the predominant source of hazardous fine particulate matter (PM_2_*_._*_5_) exposure in many parts of the western US today.^3^ It is responsible for offsetting 50% or more of the PM_2_*_._*_5_ reductions achieved in many western US states by the Clean Air Act and other policies.^4^

Despite the ubiquity of wildfire smoke exposure in the western US in the past decade, our understanding of the scope of wildfire smoke health burdens remains limited. The majority of previous wildfire epidemiological research has examined the effects of wildfire smoke exposure on mortality and respiratory and cardiovascular outcomes, finding adverse associations of wildfire smoke exposure and each of these outcomes.^5,6,7,8,9^ Some research has additionally explored connections with maternal and child health, diabetes, diarrhea, and various biomarkers.^6,10,11^ However, a broader understanding of wildfire smoke health impacts is needed to inform clinical preparedness and climate resilience-building efforts.

Recognizing the need for a more comprehensive examination of wildfire smoke health burdens, we investigated the association of smoke-specific PM_2_*_._*_5_ exposure with each of 13 causes of hospitalization among older adults in the US. Our study leveraged records for all unscheduled inpatient hospitalizations among Medicare Fee-for-Service beneficiaries aged 65 years or older in 11 western US states during 2006 to 2016. These data were linked with newly developed, spatially resolved estimates of daily surface PM_2_*_._*_5_ concentrations originating from smoke.^12^ We then assessed the associations between daily smoke PM_2_*_._*_5_ concentrations and each of 13 causes of hospitalization using panel data models that (1) allowed for nonlinear associations; (2) accounted for lagged effects of smoke; and (3) adjusted for spatial, temporal, and meteorological confounders. Compared with a recent study^13^ of associations between daily smoke PM_2.5_ and cause-specific emergency department visits in California, our work contributes evidence regarding associations between smoke PM_2.5_ and hospitalization outcomes, which are more severe and costly events that exert greater weight in policy decisions, and places a focus on vulnerable older adults across the entire western US.

## Methods

This cohort study followed the Strengthening the Reporting of Observational Studies in Epidemiology (STROBE) reporting guideline. This study was approved by the institutional review board at the Harvard T.H. Chan School of Public Health, and informed consent was waived because obtaining consent from individual Medicare beneficiaries is not required for secondary analyses of deidentified Medicare claims data.

### Study Area and Population

Our study area included 11 states in the contiguous western US (ie, Arizona, California, Colorado, New Mexico, Nevada, Utah, Montana, Idaho, Oregon, Washington, and Wyoming), which were selected due to increasing trends in PM_2_*_._*_5_ in this region.^14,15,16^ Our study period included only days during wildfire season, defined as May 1 to October 31 of each year from 2006 to 2016.^17^

Using Medicare inpatient claims data from the US Centers for Medicare & Medicaid Services, we collected data from Medicare beneficiaries aged 65 years or older who were enrolled in the Fee-For-Service program for at least 1 month during the study period and resided in the study area. For each enrollee, the county of residence was extracted from the Medicare enrollee record file, while admission date and principal *International Classification of Diseases, Ninth Revision, Clinical Modification* (*ICD-9-CM*) or *International Statistical Classification of Diseases, Tenth Revision, Clinical Modification* (*ICD-10-CM*) code for each unscheduled hospitalization were extracted from the Medicare Provider Analysis and Review file.

### Outcome Assessment

Our approach to defining hospitalization cause categories follows that of several recent studies.^18,19^ We categorized each *ICD-9-CM* and *ICD-10-CM* code using the Clinical Classifications Software (CCS) hierarchy algorithm^20^ to obtain 18 distinct and clinically meaningful level 1 disease categories. We excluded 5 categories that are infrequent among older adults, such as pregnancy or fertility issues, as well as those that were ambiguously defined. The following 13 cause categories remained: cardiovascular diseases, respiratory diseases, cancers, injuries, neuropsychiatric disorders, blood diseases, digestive system diseases, endocrine disorders, genitourinary diseases, infectious and parasitic diseases, musculoskeletal and connective tissue diseases, nervous system diseases, and skin and subcutaneous tissue diseases. For each cause of hospitalization, we constructed time series of daily admissions rates by county, which were based on enrollees’ county of residence.

For broad causes found to be associated with smoke PM_2_*_._*_5_, we further investigated associations between subcause categories, known as CCS level 3 causes, and smoke PM_2_*_._*_5_. To ensure reliability and stability of the model results, we limited this secondary analysis to subcauses that accounted for more than 25 000 hospitalizations across the study area and study period.

### Exposure Assessment

We obtained estimates of daily surface-level smoke-specific PM_2_*_._*_5_ concentrations on a 10-km grid across the contiguous US from Childs et al,^12^ which have been used in prior epidemiologic research.^13^ These were estimated from a machine learning model trained on data from ground measurements, satellite observations, and reanalysis data sources. In the western US, these smoke-specific PM_2_*_._*_5_ concentrations are expected to primarily capture wildfire smoke PM_2_*_._*_5,_ but may capture smoke PM_2_*_._*_5_ from other sources, such as prescribed burns, as well. Daily gridded exposures were aggregated to the county level for linkage with the hospitalization data using population-weighted averages (eAppendix 1 in Supplement 1).

### Covariate Data

We sourced temperature data from the Parameter-elevation Regressions on Independent Slopes Model,^21^ which compiles climate observations from monitoring networks and applies quality control procedures to create a comprehensive nationwide temperature dataset. We used gridded daily mean temperature estimates at a 4 km resolution and computed population-weighted averages to obtain county-level estimates.

### Statistical Analysis

For each cause of hospitalization separately, we fit linear regression models using a distributed lag structure on smoke PM_2.5_; that is, we model daily county-level hospitalization rates as a function of same-day smoke PM_2_*_._*_5_ exposure and exposures on each day of the preceding week. The inclusion of these lagged exposure terms allows for both health outcomes and the decision to seek treatment to manifest some time after an initial exposure. Natural cubic spline terms were placed on the smoke PM_2_*_._*_5_ exposures to allow for nonlinear associations following the findings of Heft-Neal et al.^13^ Due to the presence of some extreme and influential exposure values, county-days with smoke PM_2_*_._*_5_ greater than the 99.9th percentile (50 μg/m^3^) were excluded from the model fitting for the primary analyses. All models were adjusted for a spline on county-day mean temperature, an indicator of federal holiday status, and included fixed effects for year, county, county-month (year-agnostic), and day-of-week. Models were weighted using Medicare Fee-for-Service population size weights to account for differential stability in counties’ hospitalization rates due to population size differences. We used clustered standard errors, with counties as the unit of clustering. We report estimated concentration-response curves and 95% uniform confidence bands to characterize the change in daily cause-specific hospitalization rates associated with a range of smoke PM_2_*_._*_5_ concentrations experienced each day in the preceding week (relative to 0 smoke PM_2_*_._*_5_ each day). We conducted tests for residual autocorrelation. More details on the model specification and concentration-response curve estimation are provided in eAppendix 2 of Supplement 1.

While our estimated concentration-response curves allow for nonlinearity, we also computed on-average changes in cause-specific hospitalization rates per 10 μg/m^3^ (computed empirically by averaging the estimates of the nonlinear curves across the exposure range) for comparison with studies that estimate linear associations. To assess nonlinearity in the curves, we also estimated pointwise concentration-response curve derivatives and their 95% CIs for each outcome (eAppendix 2 of Supplement 1).

As secondary analyses, we investigated associations between smoke PM_2_*_._*_5_ and subcauses of hospitalization (with more than 25 000 hospitalizations) within each broad cause for which a significant association was found. These analyses used the same modeling approach as the broad causes. Furthermore, to compare with previous literature investigating the association between smoke PM_2_*_._*_5_ and cause-specific emergency department visits in California,^13^ we also fit models for each broad cause using only California counties.

We conducted numerous sensitivity analyses (eAppendix 3 in Supplement 2). Our primary analyses set spline knots at 10 μg/m^3^, 25 μg/m^3^, and 40 μg/m^3^, informed by the curve shapes in Heft-Neal et al.^13^ As sensitivity analyses, we assessed alternative numbers and placements of knots. We also fit models using categorized smoke PM_2_*_._*_5_ exposures. Although our primary analyses are limited to county-days with smoke PM_2.5_ of less than 50 μg/m^3^, in sensitivity analyses, we fit models to the full data without this restriction. While May to October represents the traditional western US fire season, we also conducted sensitivity analyses, including data from November and December in our models, as large fires have occurred in those months in recent years. We fit analogous distributed lag models using quasi-Poisson regression to examine sensitivity to the linear regression modeling approach.

Analyses were conducted from October 2023 to February 2025 using R version 4.3.0 (R Project for Statistical Computing)^22^ with the assistance of the R fixest package.^23^ Statistical significance was determined based on whether the null value was contained in the 95% CI.

## Results

**Figure 1.**
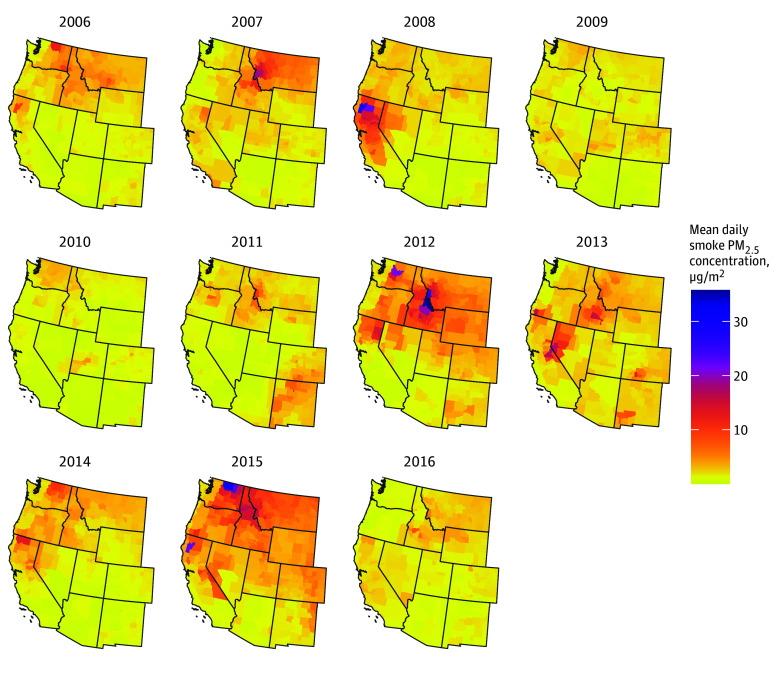
Mean Smoke-Specific Fine Particulate Matter (PM_2_*_._*_5_) During Smoke Days by County and Year in the Western US, 2006 to 2016

**Figure 2.**
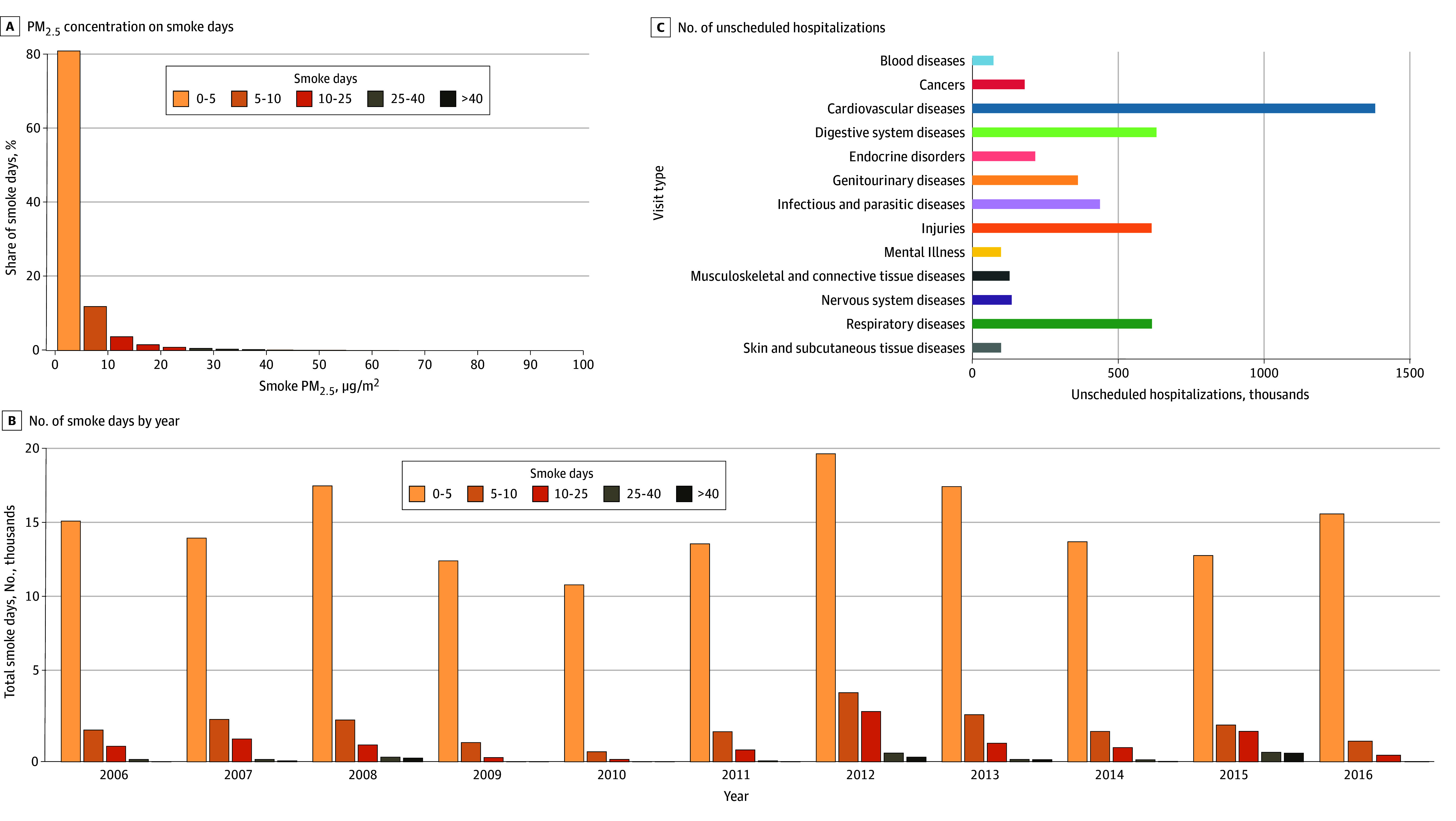
Smoke Fine Particulate Matter (PM_2_*_._*_5_) Concentration on Smoke Days, Number of Smoke Days by Year, and Number of Unscheduled Hospitalizations Among Medicare Fee-for-Service Beneficiaries Residing in the Western US During Wildfire Seasons From 2006 to 2016 (A) Histogram of smoke PM_2_*_._*_5_ (μg/m^3^) concentration on smoke days; (B) number of smoke days by year in the western US color coded by concentration of smoke PM_2_*_._*_5_ (μg/m^3^); (C) number of unscheduled hospitalizations by cause among Medicare Fee-for-Service beneficiaries residing in the western US, wildfire seasons 2006 to 2016. The western US states include Arizona, California, Colorado, New Mexico, Nevada, Utah, Montana, Idaho, Oregon, Washington, and Wyoming.

Our analyses included all 414 counties from the 11 western US states. For the purpose of generating descriptive statistics, a county-day was defined as a smoke day if the county intersected with an overhead smoke plume on that day.^12^ Across the counties in our sample, the mean (SD) number of smoke days during the study period was 498 (160). The county with the fewest smoke days during the study had 178 smoke days, and the county with the most smoke days had 894 smoke days. Figure 1 displays the distribution of the mean smoke PM_2_*_._*_5_ during smoke days across counties per year. Additionally,165 890 smoke days (80.5%) had concentrations less than 5 μg/m^3^, and an additional 24 294 smoke days (11.8%) had concentrations between 5 to 10 μg/m^3^ (Panel A in Figure 2). Smoke PM_2.5_ concentrations were between 10 to 25 μg/m^3^ on 12 445 smoke days (6%), between 25 to 40 μg/m^3^ on 2175 smoke days (1.1%), and 40 μg/m^3^ or more on 1377 smoke days (0.6%) The number of smoke days varied substantially by year with the most occurring in 2012 (Panel B in Figure 2).

The cohort included 10 369 361 individuals, of which 4 862 826 were male (46.9%), 5 506 535 were female (53.1%), 373 252 were Black (3.6%), 420 577 were Hispanic (4.1%), 8 365 607 were White (80.7%) individuals. The mean (SD) age was 74.7 (7.9) years, with 57 974 120 person-months of follow-up (eTable 2 in Supplement 1). The study included 4 760 651 unscheduled inpatient hospitalizations among 1 333 143 unique individuals. The 3 most prominent causes of unscheduled hospitalization were cardiovascular disease (mean [SD] daily rate, 7.92 [6.90] per 100 000 persons), digestive system disease (mean [SD] daily rate, 3.62 [4.39] per 100 000 persons), and respiratory disease (mean [SD] daily rate, 3.53 [4.39] per 100 000 persons) (Panel C in Figure 2 and eTable 1 in Supplement 1). The mean daily rate of unscheduled hospitalizations for each of the 13 broad cause categories can be found in eTable 1 in Supplement 1. 

**Figure 3.**
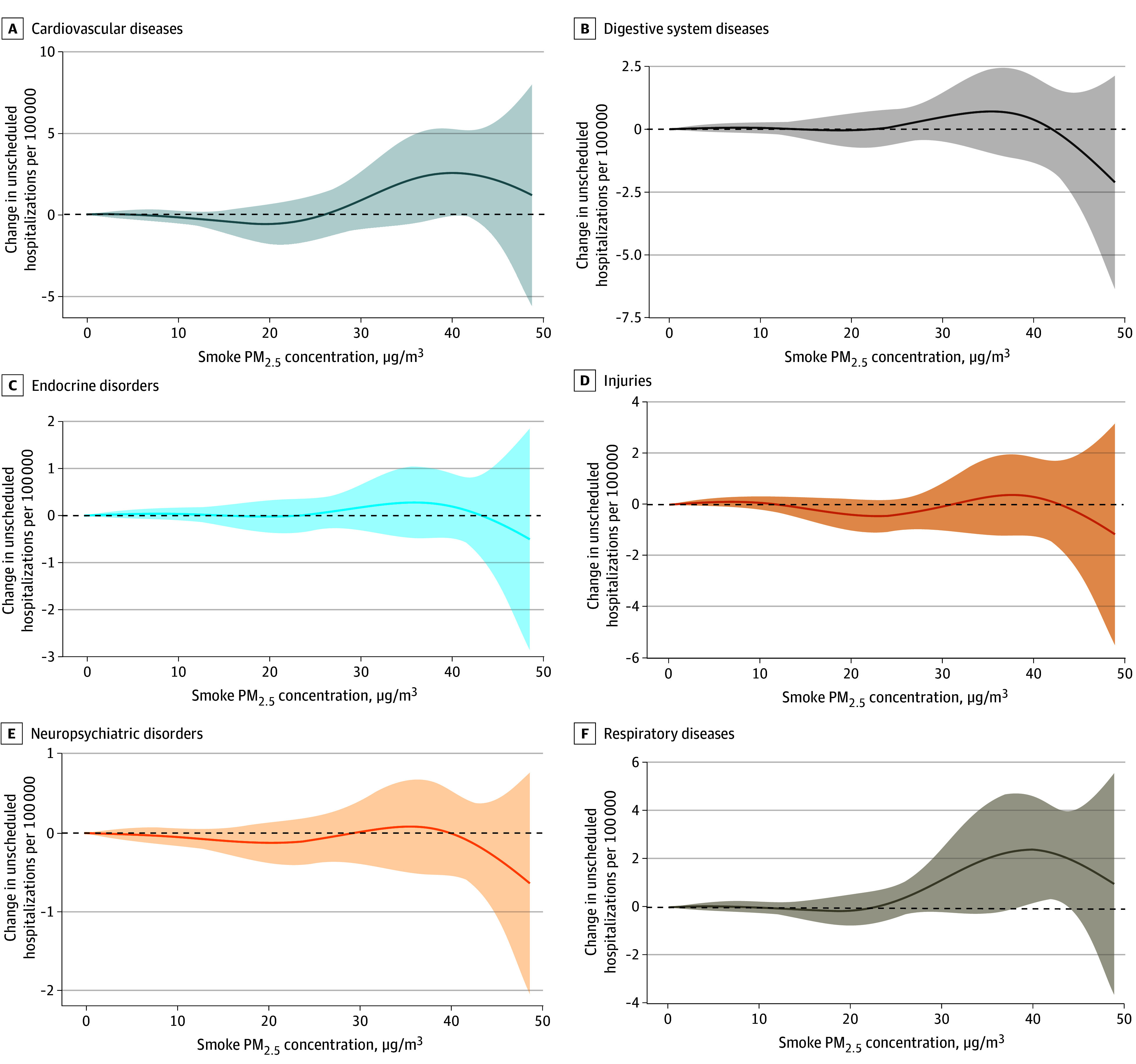
Estimated Changes in Daily Unscheduled Hospitalizations (Per 100 000) for 6 Causes Associated With a Given Smoke Fine Particulate Matter (PM_2_*_._*_5_) Concentration, Relative to 0, Experienced on the Same Day and Each of the Preceding 7 Days Lines are point estimates and shaded areas are 95% CIs, constructed using cluster robust standard errors.

The shapes of the estimated concentration-response curve for county-level smoke PM_2_*_._*_5_ and unscheduled hospitalizations varied considerably across causes (Figure 3 and eFigures 1-13, 16, and 17 in Supplement 1). These curves display the estimated change in daily, county-level hospitalization rates comparing a scenario in which a given smoke PM_2_*_._*_5_ concentration was experienced on each day in the preceding week vs a scenario with 0 smoke PM_2_*_._*_5_ on each day, and associated 95% uniform confidence bands. The mean change in hospitalization rates for each cause per 10 μg/m^3^ increase in smoke PM_2.5_ (absolute and percentage changes) are shown in eTable 4 in Supplement 1. For cardiovascular and respiratory disease hospitalizations, the concentration-response curves were generally flat at low exposure levels, began to increase around 25 μg/m^3^, and plateaued around 40 μg/m^3^ (Figure 3A and 3F). For respiratory disease hospitalizations, the confidence bands exceed 0 in parts of the exposure range, indicating a statistically significant increase in hospitalization rates at those smoke PM_2.5_ concentrations. On average, daily hospitalizations per 100 000 persons increased by 2.40 (95% CI, 0.17 to 4.63) for respiratory disease hospitalizations when increasing same-day and preceding week smoke PM_2.5_ concentrations from 0 to 40 μg/m^3^. The confidence bands for cardiovascular disease fully contain 0, but come very close to exceeding 0 at around 40 μg/m^3^. On average, daily hospitalizations per 100 000 persons increased by 2.61 (95% CI, −0.09 to 5.30) for cardiovascular hospitalizations when increasing same-day and preceding week smoke PM_2.5_ concentrations from 0 to 40 μg/m^3^. Curves for injury, neuropsychiatric, digestive system, and endocrine hospitalizations were also flat at low exposure levels but modestly decreased at high exposure levels; however the confidence bands fully contained 0. Estimated concentration-response curves were largely flat for all other causes of hospitalization. Point-wise derivatives and 95% CIs for each curve (eFigure 15 in Supplement 1) showed that curves for respiratory, cardiovascular, and injury hospitalizations have significant nonlinearity. *P* values from residual auto-correlation tests on each model are shown in eTable 3 in Supplement 1.

**Figure 4.**
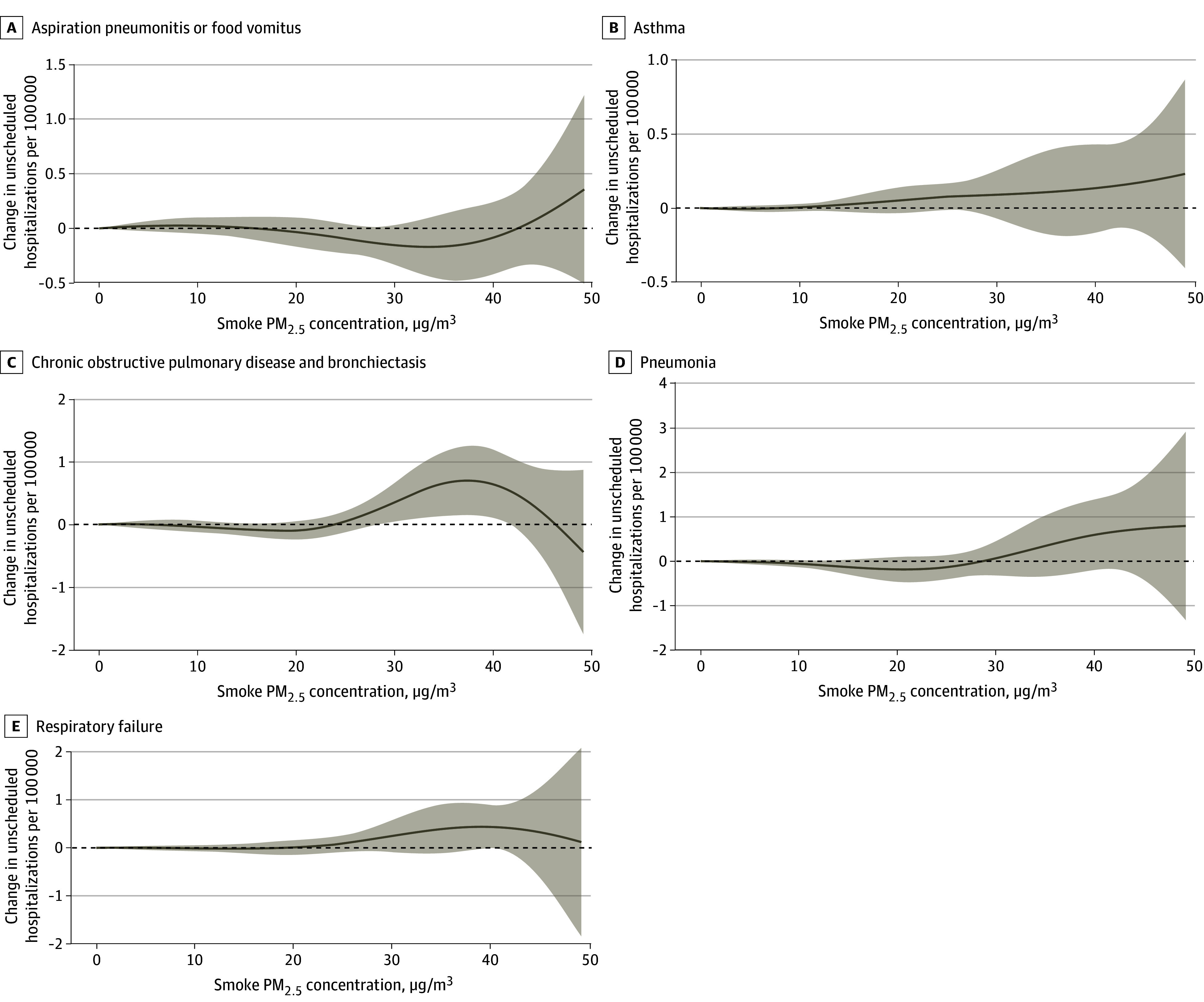
Estimated Changes in Daily Unscheduled Hospitalizations (Per 100 000) for Respiratory Subcauses Associated With a Given Smoke Fine Particulate Matter (PM_2_*_._*_5_) Concentration, Relative to 0, Experienced on the Same Day and Each of the Preceding 7 Days Lines are point estimates and shaded areas are 95% CIs, constructed using cluster robust standard errors.

Respiratory disease was the only broad cause that showed a statistically significant association with smoke PM_2_*_._*_5_, and we analyzed smoke PM_2_*_._*_5_ associations with each respiratory subcause with more than 25 000 unscheduled inpatient admissions (Figure 4). eFigure 14 in Supplement 1 shows the percentage of total respiratory hospitalizations contributed by each subcause, with pneumonia (217 546 unscheduled hospitalizations, 36.9% of all respiratory), chronic obstructive pulmonary disease (119 990 unscheduled hospitalizations, 20.4% of all respiratory), and respiratory failure (91 425 unscheduled hospitalizations, 15.5% of all respiratory) accounting for the largest proportions. Pneumonia, asthma, and respiratory failure demonstrated a similar curve shapes to the overall respiratory curve. The concentration-response curve for chronic obstructive pulmonary disease hospitalization was flat below 20 μg/m^3^, with an increased risk of hospitalization between 20 to 40 μg/m^3^ followed by a decreasing risk above 40 μg/m^3^. However, confidence bands were wide above 40 μg/m^3.^

When analyzing California data only (Panel C in eFigures 1 to 13 in Supplement 1), a significant positive association was observed for cardiovascular hospitalizations at high smoke PM_2.5_ concentrations. Meanwhile stronger, but not statistically significant, decreases were observed at high smoke PM_2.5_ concentrations for nervous system and infectious disease hospitalizations.

In sensitivity analyses with alternate spline knot placements and categorized smoke PM_2_*_._*_5_ exposures, overall patterns and conclusions were found to be robust (Panels F-H in eFigures 1-13 in Supplement 1). In spline models fitted without restricting the exposure range, the shapes of the curves below 50 μg/m^3^ for many causes changed (Panels D and E in eFigures 1-13 in Supplement 1). These discrepancies are likely partly due to the extreme values of a small number of observed exposures above 50 μg/m^3^ (0.01% of county-days), which have large influence and lead to instability in the curves. When the binned exposure models were fitted with or without restricting the exposure range (Panels K and L in eFigures 1-13 in Supplement 1), patterns were generally consistent with our primary results. Conclusions from sensitivity analyses including data from November to December were consistent with the main analyses (Panel I in eFigures 1-13 in Supplement 1). Curves estimated via quasi-Poisson regression also generally resembled those from the main analyses (Panel J in eFigures 1-13 in Supplement 1).

## Discussion

This study, to our knowledge the largest study of smoke PM_2_*_._*_5_ and hospitalization to date, found strong evidence of a nonlinear association between daily, county-level smoke PM_2_*_._*_5_ and respiratory hospitalizations and weaker but suggestive evidence for cardiovascular hospitalizations. In our respiratory and cardiovascular analyses, hospitalization rates were largely unrelated to smoke PM_2_*_._*_5_ at levels less than 20 μg/m^3^, with a positive association observed at higher concentrations. For several other causes (injuries, neuropsychiatric disorders, and digestive system diseases), we observed modest, nonsignificant decreases in hospitalization rates associated with high smoke PM_2_*_._*_5_.

Nonlinearity in total PM_2.5_ concentration-response curves for certain outcomes, which has been widely reported,^24,25,26,27,28,29^ is often attributed to variability in particle compositions. Variability in particle composition may also partly explain nonlinear associations for smoke PM_2.5_. Recent literature has also identified biological mechanisms that may underpin nonlinearity in PM_2.5_-health associations.^30^ However, the nonlinearity detected in our estimated smoke PM_2.5_ concentration-response curves is likely due, at least in part, to health-protective behavioral changes that take place when the public is aware that smoke pollution concentrations are high. Previous studies have found that many individuals limit outdoor activity and seek information about protection from poor air quality during wildfire smoke episodes.^13,31^ This may explain the leveling off or decreases in curves at high concentrations. This phenomenon may also complicate comparisons of estimated toxicity of smoke PM_2.5_ relative to total PM_2.5_.

Previous wildfire smoke epidemiologic studies have emerged primarily from the US and Australia and have largely investigated only a single year, state, or wildfire event. These studies have consistently observed associations between wildfire smoke and respiratory hospitalizations, while the evidence for associations with cardiovascular hospitalizations has been mixed.^6,7,8^ For respiratory, numerous studies have found strong positive associations with respiratory disease hospitalizations overall and with subcauses including asthma, chronic obstructive pulmonary disease, and pneumonia.^32,33,34,35,36,37,38,39,40^ Our findings, representing a larger and more diverse population, reinforce these previous findings and provide further insights about the shapes of concentration-response curves for respiratory hospitalization and smoke PM_2_*_._*_5_. Our estimated curve was flat below 25 μg/m^3^ with an increasing trend between 25 to 40 μg/m^3^, and an apparent leveling off between 40 to 50 μg/m^3^. Evidence of similar nonlinearity was reported by Liu et al.^32^

The association between wildfire smoke exposure and cardiovascular hospitalization is less well established, with previous research yielding mixed results. Most studies have found no association between wildfire smoke exposure and cardiovascular hospitalization overall,^32,34,35,37,38,39^ although a few have found positive associations.^40,41^ Mixed findings have also been reported for cardiovascular subcauses.^33,34,37,39,42^ Our findings offer weak but suggestive evidence of a nonlinear association between cardiovascular disease hospitalizations and smoke PM_2_*_._*_5_ in older adults in the US, with curve shape and magnitude similar to those for respiratory hospitalizations but wider confidence bands.

To our knowledge, only one prior study by Heft-Neal et al^13^ has undertaken a comparably wide-ranging investigation of smoke PM_2_*_._*_5_ health burdens, focused on associations with cause-specific zip code-level emergency department visit rates in California from 2006 to 2017. They found that respiratory emergency department visits consistently increased with smoke PM_2_*_._*_5_. In contrast, visits for other causes increased at lower concentrations of smoke PM_2_*_._*_5_ but decreased at higher concentrations. Further investigation suggested that this decrease was attributable to health-protective behavioral responses to extreme smoke pollution. Here, in analyses of the entire western US and restricted to California, we only observed modest evidence of decreases in hospitalization rates at high smoke PM_2_*_._*_5_ concentrations for a few causes. This may suggest that hospitalization outcomes are less dramatically impacted by behavioral modifications than emergency department visits.

### Strengths and Limitations

Strengths of this work include the large population and long study period and the use of new machine learning–derived smoke-specific PM_2_*_._*_5_ exposure estimates. However, this study has limitations. Despite reliance on state-of-the-art exposure measures, exposure measurement error is likely still present. Smoke-specific PM_2_*_._*_5_ concentrations are challenging to estimate because they are not directly measured. Moreover, because intra-county pollution concentrations can vary considerably, county-level exposure measures are an imperfect proxy of the exposures experienced by residents of the county. The resulting exposure misclassification could bias estimated associations; yet it would likely result in bias toward the null.^43^ Our use of a time series analysis, which accounts for county-level time-invariant confounders through fixed effects and adjusts for numerous time-varying factors, is a strength; however, we cannot rule out the possibility of residual confounding. Because our data extend only through 2016, we were unable to incorporate information from more recent years during which western US wildfires have intensified. Additionally, due to the sparsity of county-level exposures greater than 50 μg/m^3^, we were unable to reliably characterize the associations at higher concentrations. Further research is needed to understand the effect of these extreme events.

## Conclusions

In this cohort study of older adults in the US, exposure to high levels of smoke pollution was associated with an increase in hospitalizations for respiratory diseases. Findings for cardiovascular disease were not statistically significant, but suggest the need for further investigation. This information can be used by both policymakers and clinicians to design policies and guidelines to protect vulnerable older adults from the escalating health threats posed by wildfire smoke.

## Notes

Supplement 1. **eAppendix 1.** Smoke PM_2.5_ exposure **eAppendix 2.** Statistical models **eAppendix 3.** Sensitivity analyses **eFigure 1.** Changes in daily unscheduled hospitalizations per 100 000 as smoke PM_2.5_ concentration increases for cardiovascular disease **eFigure 2.** Changes in daily unscheduled hospitalizations per 100 000 as smoke PM_2.5_ concentration increases for respiratory disease **eFigure 3.** Changes in daily unscheduled hospitalizations per 100 000 as smoke PM_2.5_ concentration increases for cancer **eFigure 4.** Changes in daily unscheduled hospitalizations per 100 000 as smoke PM2.5 concentration increases for injury **eFigure 5.** Changes in daily unscheduled hospitalizations per 100 000 as smoke PM_2_*_._*_5_ concentration increases for neuropsychiatric disorders **eFigure 6.** Changes in daily unscheduled hospitalizations per 100 000 as smoke PM_2_*_._*_5_ concentration increases for blood diseases **eFigure 7.** Changes in daily unscheduled hospitalizations per 100 000 as smoke PM_2_*_._*_5_ concentration increases for digestive system diseases **eFigure 8.** Changes in daily unscheduled hospitalizations per 100 000 as smoke PM_2_*_._*_5_ concentration increases for endocrine disorders **eFigure 9.** Changes in unscheduled hospitalizations per 100 000 as smoke PM_2_*_._*_5_ concentration increases for genitourinary diseases **eFigure 10.** Changes in daily unscheduled hospitalizations per 100 000 as smoke PM_2_*_._*_5_ concentration increases for infectious and parasitic diseases **eFigure 11.** Changes in daily unscheduled hospitalizations per 100 000 as smoke PM_2_*_._*_5_ concentration increases for musculoskeletal and connective tissue diseases **eFigure 12.** Changes in daily unscheduled hospitalizations per 100 000 as smoke PM_2_*_._*_5_ concentration increases for nervous system diseases **eFigure 13.** Changes in daily unscheduled hospitalizations per 100 000 as smoke PM_2_*_._*_5_ concentration increases for skin and subcutaneous tissue diseases **eFigure 14.** Percentage of total unscheduled hospitalizations each subcause contributes to the respiratory disease broad cause **eFigure 15.** Point-wise derivatives of the estimated concentration-response curves for each outcome, and their 95% CIs **eFigure 16.** Main model results plotted on the same y-axis **eFigure 17.** Estimated changes in daily unscheduled hospitalizations (per 100 000) for 13 causes associated with a given smoke PM_2.5_ concentration, relative to 0, experienced on the same day and each of the preceding 7 days **eTable 1.** Average daily rate of hospitalization (per 100 000 population) for each cause among the study population and time-period **eTable 2.** Summary demographic information **eTable 3.** The *P* values from tests of residual autocorrelation for the primary model for each cause of hospitalization **eTable 4.** On-average change in rates of hospitalization for each cause (on both absolute and percent change scales) per 10 µg/m3 increase in smoke PM_2.5_ across the exposure range computed empirically from the nonlinear curve estimates in Figure 3

Supplement 2.
Data Sharing Statement
