# Supplementary material for: Wildfire Smoke Exposure and Cause-Specific Hospitalization in Older Adults
Source: JAMA Netw Open. 2025 Apr 30;8(4):e257956. doi: 10.1001/jamanetworkopen.2025.7956 (PMC12044514; doi:10.1001/jamanetworkopen.2025.7956)
Supplement: Supplement 1. — eAppendix 1. Smoke PM2.5 exposure eAppendix 2. Statistical models eAppendix 3. Sensitivity analyses eFigure 1. Changes in daily unscheduled hospitalizations per 100 000 as smoke PM2.5 concentration increases for cardiovascular disease eFigure 2. Changes in daily unscheduled hospitalizations per 100 000 as smoke PM2.5 concentration increases for respiratory disease eFigure 3. Changes in daily unscheduled hospitalizations per 100 000 as smoke PM2.5 concentration increases for cancer eFigure 4. Changes in daily unscheduled hospitalizations per 100 000 as smoke PM2.5 concentration increases for injury eFigure 5. Changes in daily unscheduled hospitalizations per 100 000 as smoke PM2.5 concentration increases for neuropsychiatric disorders eFigure 6. Changes in daily unscheduled hospitalizations per 100 000 as smoke PM2.5 concentration increases for blood diseases eFigure 7. Changes in daily unscheduled hospitalizations per 100 000 as smoke PM2.5 concentration increases for digestive system diseases eFigure 8. Changes in daily unscheduled hospitalizations per 100 000 as smoke PM2.5 concentration increases for endocrine disorders eFigure 9. Changes in unscheduled hospitalizations per 100 000 as smoke PM2.5 concentration increases for genitourinary diseases eFigure 10. Changes in daily unscheduled hospitalizations per 100 000 as smoke PM2.5 concentration increases for infectious and parasitic diseases eFigure 11. Changes in daily unscheduled hospitalizations per 100 000 as smoke PM2.5 concentration increases for musculoskeletal and connective tissue diseases eFigure 12. Changes in daily unscheduled hospitalizations per 100 000 as smoke PM2.5 concentration increases for nervous system diseases eFigure 13. Changes in daily unscheduled hospitalizations per 100 000 as smoke PM2.5 concentration increases for skin and subcutaneous tissue diseases eFigure 14. Percentage of total unscheduled hospitalizations each subcause contributes to the respiratory disease broad cause eFigure 1 [file jamanetwopen-e257956-s001.pdf]

Supplemental Online Content

Vega SL, Childs ML, Aggarwal S, Nethery RC. Wildfire smoke exposure and cause-specific hospitalization in older adults. *JAMA Netw Open*. 2025;8(4):e257956. doi:10.1001/jamanetworkopen.2025.7956

eAppendix 1. Smoke $PM_{2.5}$ exposure
eAppendix 2. Statistical models
eAppendix 3. Sensitivity Analyses
eFigure 1. Changes in daily unscheduled hospitalizations per 100,000 as smoke PM2.5 concentration increases for cardiovascular disease
eFigure 2. Changes in daily unscheduled hospitalizations per 100,000 as smoke PM2.5 concentration increases for respiratory disease
eFigure 3. Changes in daily unscheduled hospitalizations per 100,000 as smoke $PM_{2.5}$ concentration increases for cancer
eFigure 4. Changes in daily unscheduled hospitalizations per 100,000 as smoke PM2.5 concentration increases for injury
eFigure 5. Changes in daily unscheduled hospitalizations per 100,000 as smoke $PM_{2.5}$ concentration increases for neuropsychiatric disorders
eFigure 6. Changes in daily unscheduled hospitalizations per 100,000 as smoke $PM_{2.5}$ concentration increases for blood diseases
eFigure 7. Changes in daily unscheduled hospitalizations per 100,000 as smoke $PM_{2.5}$ concentration increases for digestive system diseases
eFigure 8. Changes in daily unscheduled hospitalizations per 100,000 as smoke $PM_{2.5}$ concentration increases for endocrine disorders
eFigure 9. Changes in unscheduled hospitalizations per 100,000 as smoke $PM_{2.5}$ concentration increases for genitourinary diseases
eFigure 10. Changes in daily unscheduled hospitalizations per 100,000 as smoke $PM_{2.5}$ concentration increases for infectious and parasitic diseases
eFigure 11. Changes in daily unscheduled hospitalizations per 100,000 as smoke $PM_{2.5}$ concentration increases for musculoskeletal and connective tissue diseases
eFigure 12. Changes in daily unscheduled hospitalizations per 100 000 as smoke $PM_{2.5}$ concentration increases for nervous system diseases
eFigure 13. Changes in daily unscheduled hospitalizations per 100,000 as smoke $PM_{2.5}$ concentration increases for skin and subcutaneous tissue diseases
eFigure 14. Percentage of total unscheduled hospitalizations each sub-cause contributes to the respiratory disease broad cause
eFigure 15. Point-wise derivatives of the estimated concentration-response curves for each outcome, and their 95% CIs
eFigure 16. Main model results plotted on the same y-axis
eFigure 17. Estimated changes in daily unscheduled hospitalizations (per 100,000) for 13 causes associated with a given smoke PM2.5 concentration, relative to 0, experienced on the same day and each of the preceding 7 days
eTable 1. Average daily rate of hospitalization (per 100,000 population) for each cause among the study population and time-period
eTable 2. Summary demographic information
eTable 3. The *P* values from tests of residual autocorrelation for the primary model for each cause of hospitalization
eTable 4. On-average change in rates of hospitalization for each cause (on both absolute and percent change scales) per 10 μg/m3 increase in smoke $PM_{2.5}$ across the exposure range computed empirically from the nonlinear curve estimates in Figure 3

This supplemental material has been provided by the authors to give readers additional information about their work.

© 2025 Vega SL et al. *JAMA Network Open*.

eAppendix 1. Smoke $PM_{2.5}$ exposure

In Childs et al (2022), daily smoke PM2.5 concentrations were originally predicted across a 10km grid. The same team also provided the county-level smoke PM2.5 exposure estimates used in our analyses, which were created via population-weighted averaging of the 10km gridded concentrations. We summarize the procedure for creating those estimates, per the publicly-available code used (https://github.com/echolab-stanford/daily-10km-smokePM). Specifically, WorldPop population density data were collected for each 10km grid cell. For each county, each 10km grid cell intersecting the county was identified and its area of overlap with the county was computed. The population size within each grid cell's overlapping area was computed by multiplying the grid cell's population density by the overlap area. Finally, the county's population-weighted average exposure was estimated by computing a weighted average of the exposure in each grid cell overlapping the county, weighted by the population in the grid cell's overlapping area.

eAppendix 2. Statistical models

In the primary analyses, for each cause of hospitalization the mean structure of the model was specified as:

$$E[y_{cd}] = \sum_{l=0}^{7} \beta_l' \; f\left(smoke_{c,d-l}\right) + \alpha' \; g\left(TEMP_{cd}\right) + \gamma \; HOLIDAY_d + \delta_{year} + \theta_c + \omega_{cm} + \eta_{dow} \qquad (1)$$

where y_{cd} is the rate of hospitalizations per 100,000 at county c and day d and δ_{year}, θ_c, ω_{cm}, and η_{dow} are fixed effects for year, county, county-month, and day-of-week, respectively. For each of day d and the seven prior "lag" days (days $d - l$ for l = 0, ..., 7), $f\left(smoke_{c,d-l}\right)$ is a spline basis applied to the smoke $PM_{2.5}$ exposure in county c and day $d - l$. The β_l represent vectors of lag day-specific smoke $PM_{2.5}$ spline basis function coefficients. $TEMP_{cd}$ is the mean temperature at county c and day d and $g(TEMP_{cd})$ is a spline basis applied to temperature. $HOLIDAY_d$ is a binary indicator of whether day d was a federal holiday. α and γ are the coefficients for these adjustment variables.

To derive the cumulative effect of smoke $PM_{2.5}$ exposure on cause-specific hospitalizations over eight days (lags 0–7), we first summed the lag specific coefficient vectors element-wise to obtain a vector $\hat{\phi}$, i.e., $\hat{\phi} = \sum_{l=0}^{7} \hat{\beta}_l$. We then created a grid of possible smoke $PM_{2.5}$ concentration values across the range 0-49 μg/m^3. The

curves shown in Figure 3 were constructed point-wise by evaluating the spline basis function for each grid point and then taking the inner product of the spline basis and ϕ. That is, for C = smoke $PM_{2.5}$ concentration from 0 to 49 μg/m^3, we plot $\left(C,\ \widehat{\phi'} f(C)\right)$. The 95% confidence intervals for the curves in Figure 3 were obtained using cluster robust standard errors, with counties as clusters.

To assess the potential nonlinearity in the estimated concentration-response curves, we estimated the pointwise first derivatives of the concentration-response curve for each health outcome and the corresponding 95% confidence intervals. The derivative quantifies the rate of change of the estimated concentration-response curve at each point on the curve. Significant non-linearity in a given curve can be identified by examining whether there are exposure levels for which the 95% confidence intervals of the derivatives do not overlap, indicating significantly differing rates of change of the curve at different exposure levels.

In the sensitivity analysis using categorized exposures, the mean structure of the model was specified as:

$$E[y_{cd}] = \sum_{b=1}^{B}\left(\sum_{l=0}^{7} \beta_{b,l}\ smokeBIN_{\ c,d-l}^{\ b}\right) + \alpha X_{cd} + \delta_{year} + \theta_c + \omega_{cm} + \eta_{dow} \quad (2)$$

with the same fixed effects and control for mean temperature. Here, the spline term from Equation 1 is replaced with the indicator variable $smokeBIN_{c,d-l}^{b}$ for whether the wildfire smoke $PM_{2.5}$ concentration for county *c* on day *d* – *l* falls within the range of bin *b*. We divide $PM_{2.5}$ concentrations into bins from 0-5, 5-10, 10-25, 25-40, and 40+ μg/m^3. Point estimates for each bin represent the cumulative effects across all lags. That is, we sum the coefficients for each bin across all lags, $\hat{\phi} = \sum_{l=0}^{7} \widehat{\beta_{b,l}}$. We then used the cluster standard errors from the model to calculate the standard errors and confidence intervals for the cumulative effect.

eAppendix 3. Sensitivity Analyses

We conducted a number of sensitivity analyses. Because splines can be sensitive to the number and placement of knots, we assessed models using several different knot selections. Our primary analyses used knots at 10, 25, and 40 μg/m^3. As sensitivity analyses, we also assessed models with knots placed at 5, 10, 25 μg/m^3, 5, 20, 35 μg/m^3, and 5, 10, 25, and 40 μg/m^3. We also fitted models using categorized smoke $PM_{2.5}$

© 2025 Vega SL et al. *JAMA Network Open*.

exposures instead of splines, with daily smoke $PM_{2.5}$ concentrations grouped into the following five categories (in $\mu g/m^3$): 0, (0,5], (5,10], (10,25], (25,40], > 40. To evaluate how a small number of observations with extremely high smoke $PM_{2.5}$ might influence the shape of the estimated dose-response curves, we fitted the models to the full data without excluding county-days above 50 $\mu g/m^3$. While May-October represents the traditional fire season in the Western U.S., we also conducted sensitivity analyses including data from November and December in our models, as several large fires have occurred in those months in recent years. To examine sensitivity to the linear regression modeling approach, we fit distributed lag models using quasi-Poisson regression, with all other model specifications analogous to those for the main analysis.

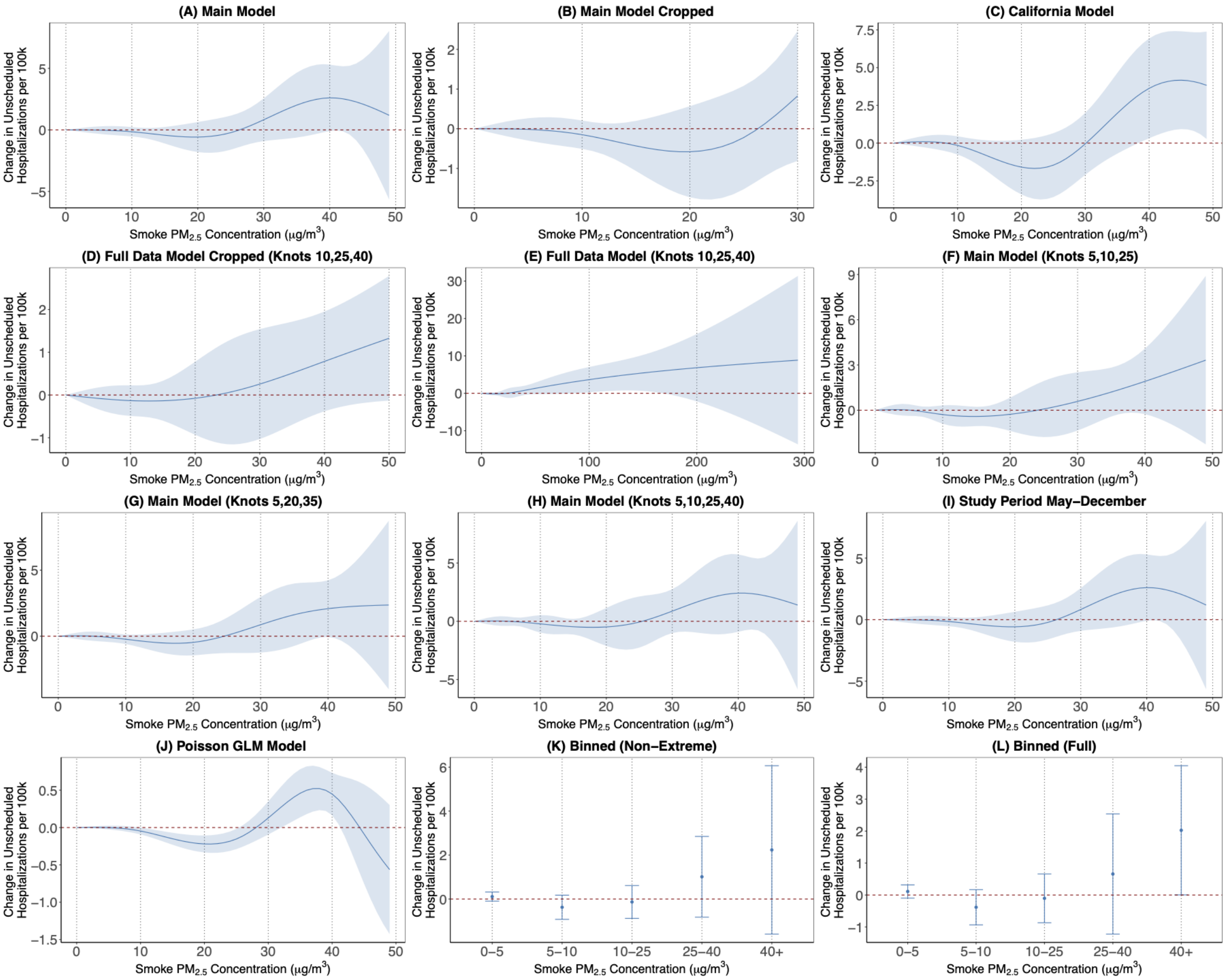

eFigure 1: Changes in daily unscheduled hospitalizations per 100,000 as smoke $PM_{2.5}$ concentration increases for cardiovascular disease

(A) Main model. (B) Main model with x-axis truncated at 30μg/m^3. (C) Main model fit on California. (D) Spline model fit on the full range of smoke $PM_{2.5}$ values with knots at 10, 25, and 40μg/m^3 and x-axis truncated at 50μg/m^3. (E) Spline model fit on the full range of smoke $PM_{2.5}$ values with knots at 10, 25, and 40μg/m^3. (F) Spline model with knots at 5, 10, 25μg/m^3. (G) Spline model with knots at 5, 20, 35μg/m^3. (H) Spline model with knots at 5, 10, 25, 40μg/m^3. (I) Main spline model with study period from May to December. (J) Poisson GLM model. (K) Binned model. (L) Binned model fit on full range of smoke $PM_{2.5}$ values.

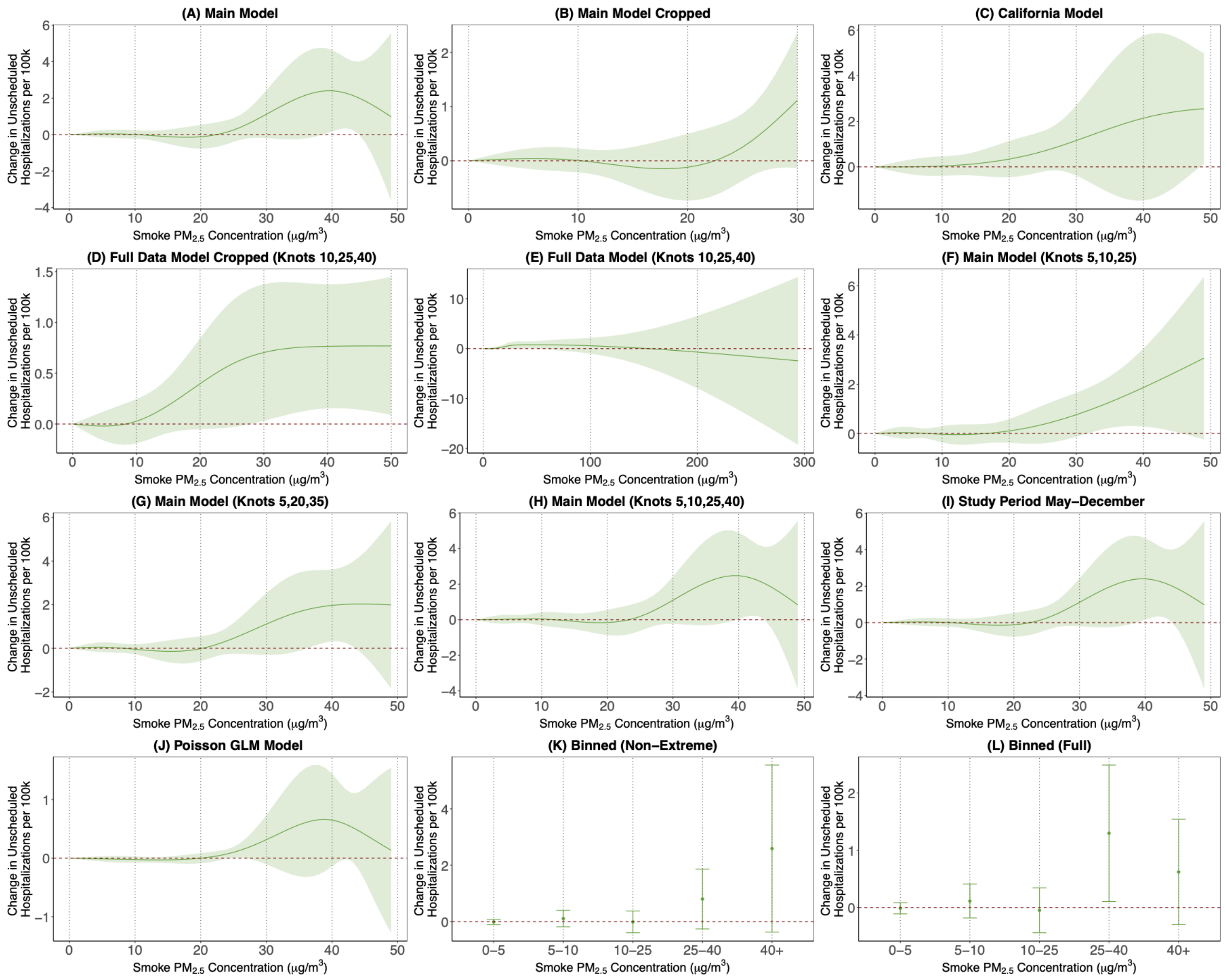

eFigure 2. Changes in daily unscheduled hospitalizations per 100,000 as smoke $PM_{2.5}$ concentration increases for respiratory disease

© 2025 Vega SL et al. *JAMA Network Open*.

(A) Main model. (B) Main model with x-axis truncated at 30μg/m^3. (C) Main model fit on California. (D) Spline model fit on the full range of smoke $PM_{2.5}$ values with knots at 10, 25, and 40μg/m^3 and x-axis truncated at 50μg/m^3. (E) Spline model fit on the full range of smoke $PM_{2.5}$ values with knots at 10, 25, and 40μg/m^3. (F) Spline model with knots at 5, 10, 25μg/m^3. (G) Spline model with knots at 5, 20, 35μg/m^3. (H) Spline model with knots at 5, 10, 25, 40μg/m^3. (I) Main spline model with study period from May to December. (J) Poisson GLM model. (K) Binned model. (L) Binned model fit on full range of smoke $PM_{2.5}$ values.

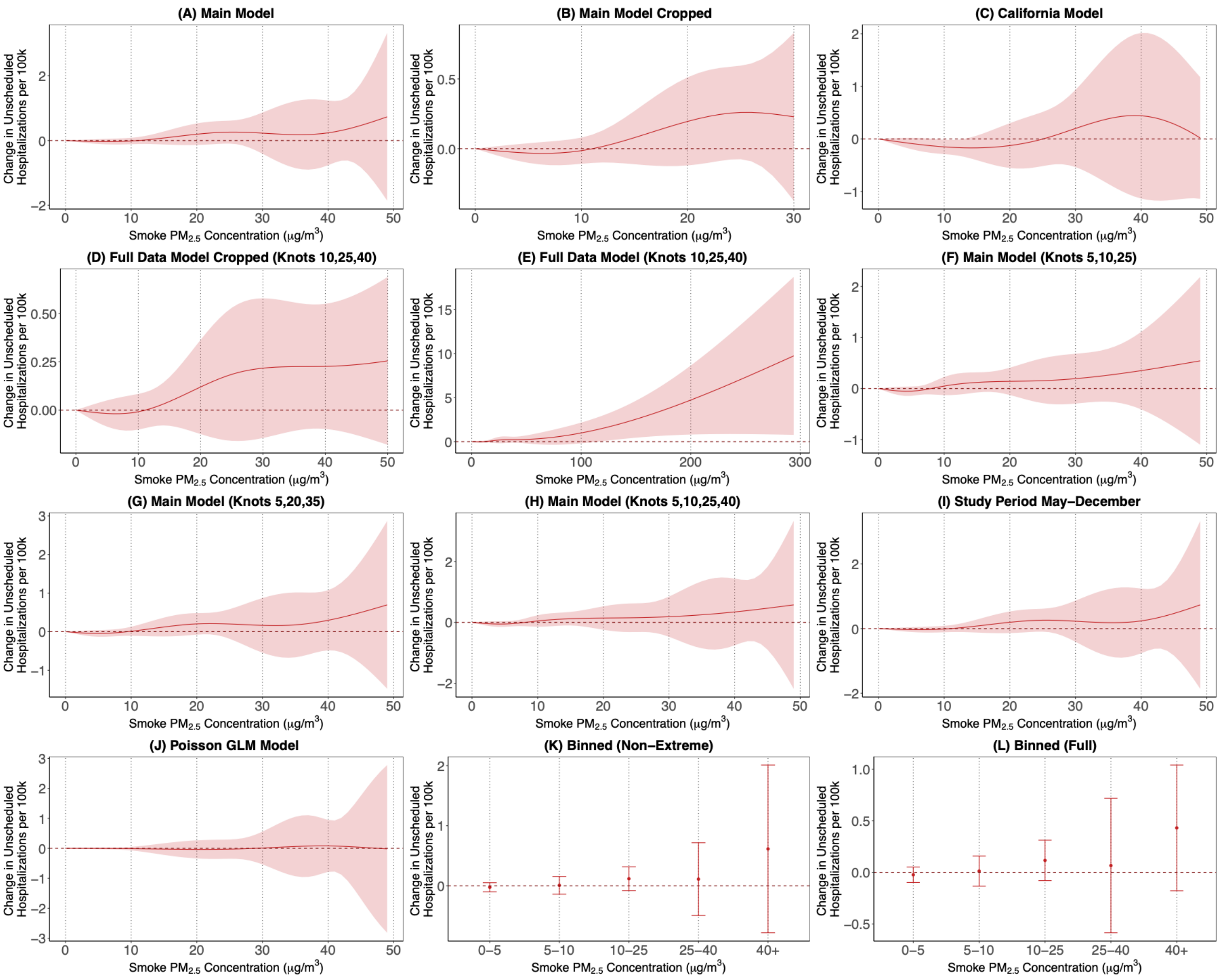

eFigure 3. Changes in daily unscheduled hospitalizations per 100,000 as smoke $PM_{2.5}$ concentration increases for cancer

(A) Main model. (B) Main model with x-axis truncated at 30$\mu g/m^3$. (C) Main model fit on California. (D) Spline model fit on the full range of smoke $PM_{2.5}$ values with knots at 10, 25, and 40$\mu g/m^3$ and x-axis truncated at 50$\mu g/m^3$. (E) Spline model fit on the full range of smoke $PM_{2.5}$ values with knots at 10, 25, and 40$\mu g/m^3$. (F) Spline model with knots at 5, 10, 25$\mu g/m^3$. (G) Spline model with knots at 5, 20, 35$\mu g/m^3$. (H) Spline model with knots at 5, 10, 25, 40$\mu g/m^3$. (I) Main spline model with study period from May to December. (J) Poisson GLM model. (K) Binned model. (L) Binned model fit on full range of smoke $PM_{2.5}$ values.

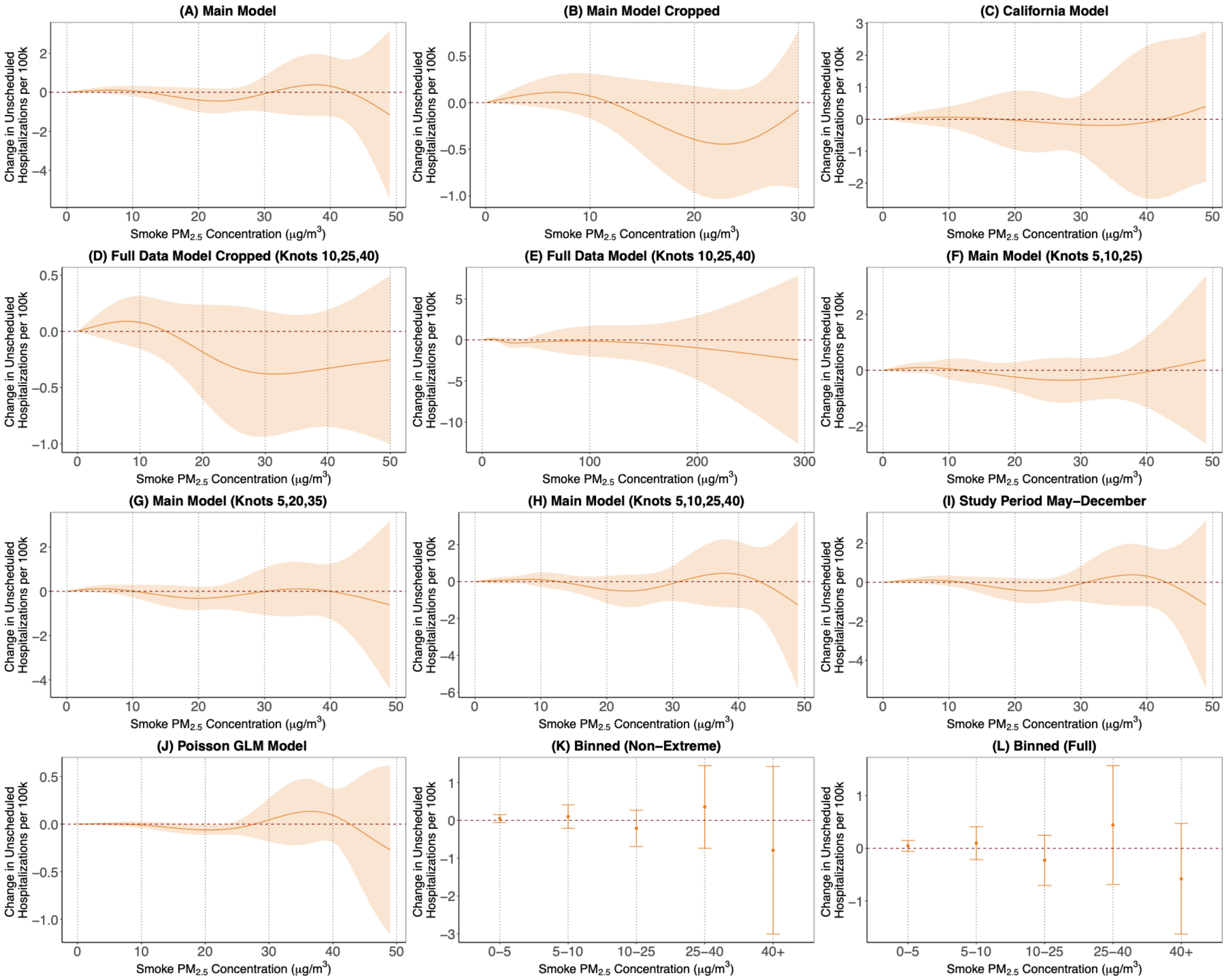

eFigure 4: Changes in daily unscheduled hospitalizations per 100,000 as smoke $PM_{2.5}$ concentration increases for injury

© 2025 Vega SL et al. *JAMA Network Open*.

(A) Main model. (B) Main model with x-axis truncated at 30$\mu g/m^3$. (C) Main model fit on California. (D) Spline model fit on the full range of smoke $PM_{2.5}$ values with knots at 10, 25, and 40$\mu g/m^3$ and x-axis truncated at 50$\mu g/m^3$. (E) Spline model fit on the full range of smoke $PM_{2.5}$ values with knots at 10, 25, and 40$\mu g/m^3$. (F) Spline model with knots at 5, 10, 25$\mu g/m^3$. (G) Spline model with knots at 5, 20, 35$\mu g/m^3$. (H) Spline model with knots at 5, 10, 25, 40$\mu g/m^3$. (I) Main spline model with study period from May to December. (J) Poisson GLM model. (K) Binned model. (L) Binned model fit on full range of smoke $PM_{2.5}$ values.

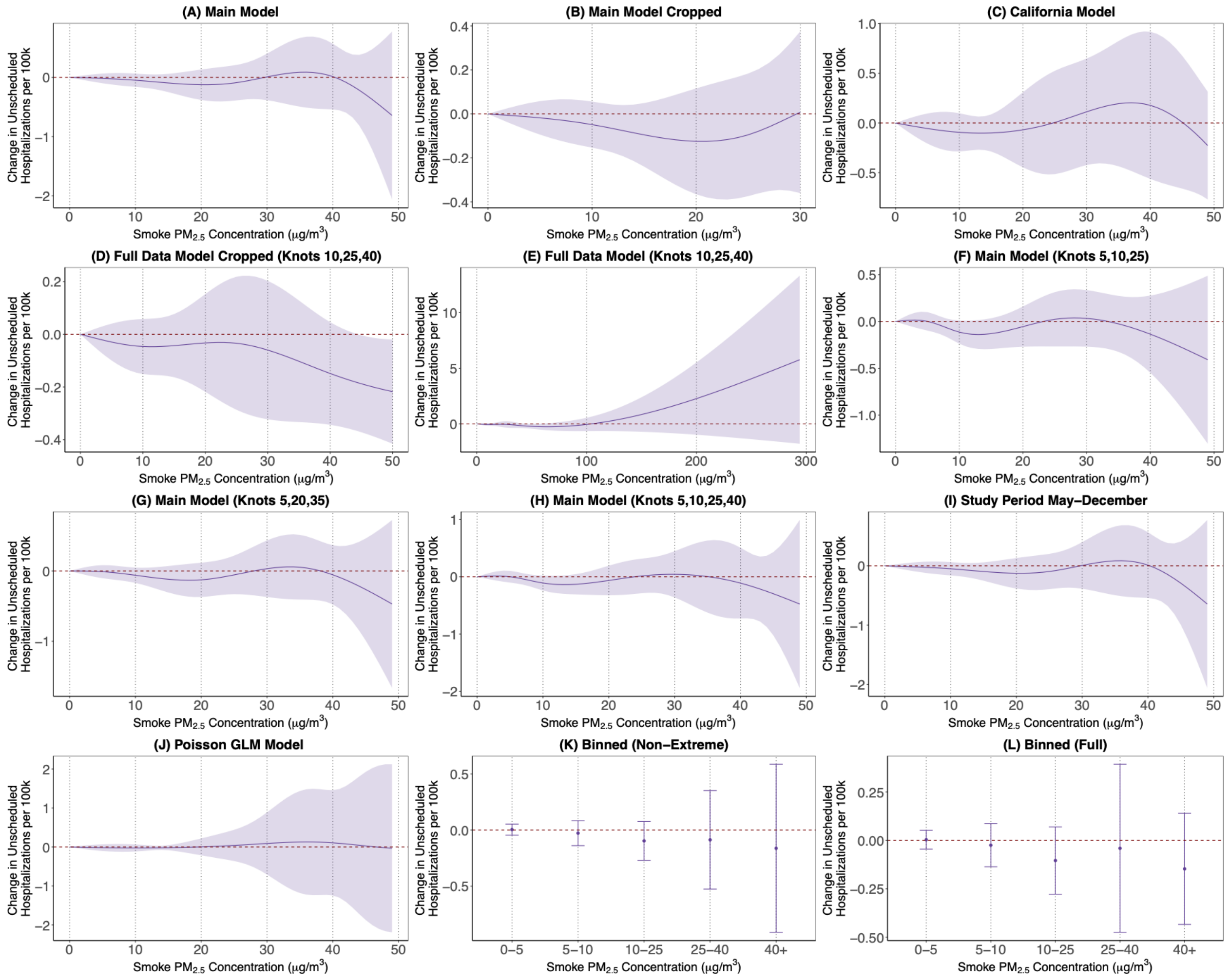

eFigure 5: Changes in daily unscheduled hospitalizations per 100,000 as smoke $PM_{2.5}$ concentration increases for neuropsychiatric disorders

(A) Main model. (B) Main model with x-axis truncated at 30μg/m^3. (C) Main model fit on California. (D) Spline model fit on the full range of smoke $PM_{2.5}$ values with knots at 10, 25, and 40μg/m^3 and x-axis truncated at 50μg/m^3. (E) Spline model fit on the full range of smoke $PM_{2.5}$ values with knots at 10, 25, and 40μg/m^3. (F) Spline model with knots at 5, 10, 25μg/m^3. (G) Spline model with knots at 5, 20, 35μg/m^3. (H) Spline model with knots at 5, 10, 25, 40μg/m^3. (I) Main spline model with study period from May to December. (J) Poisson GLM model. (K) Binned model. (L) Binned model fit on full range of smoke $PM_{2.5}$ values.

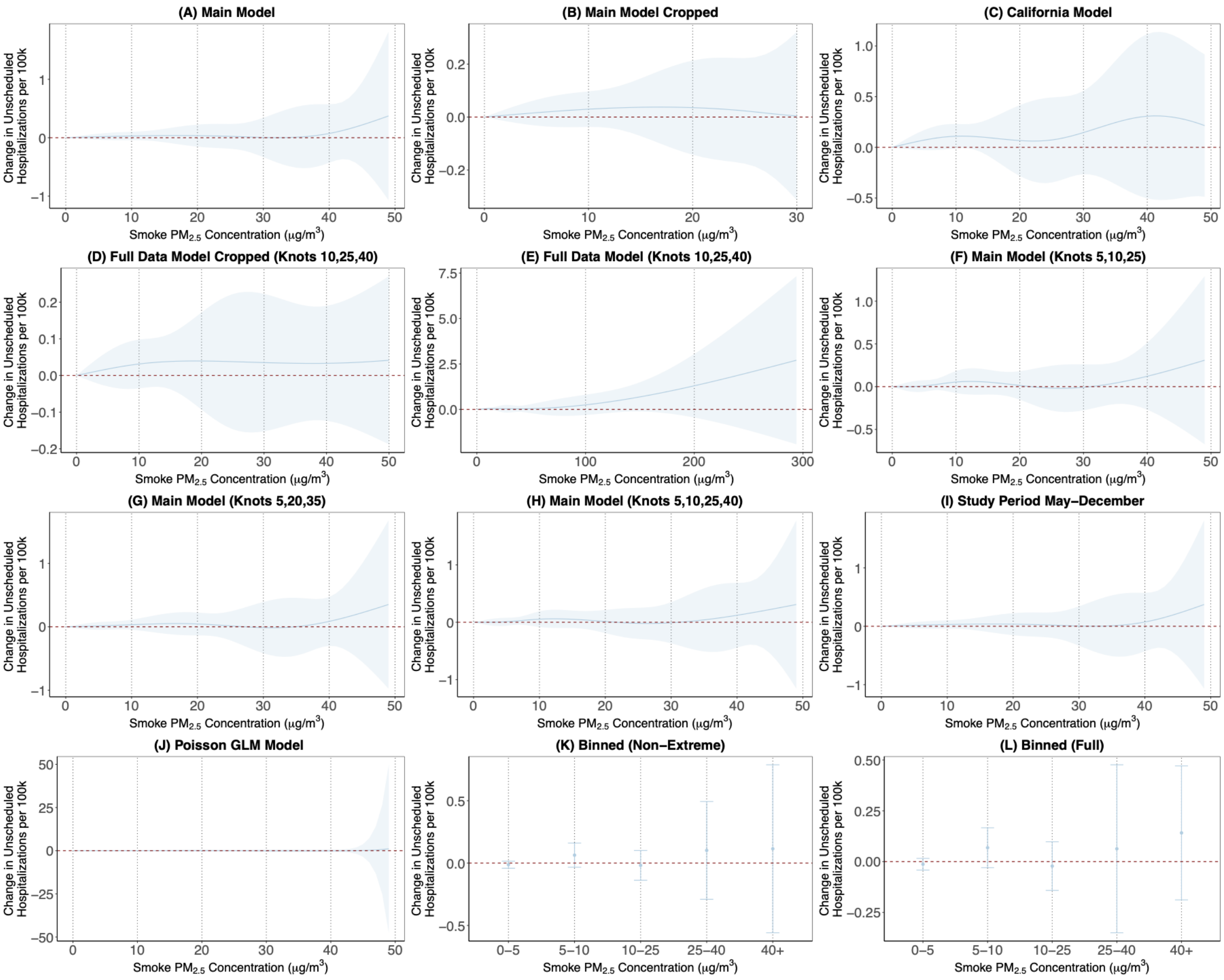

eFigure 6. Changes in daily unscheduled hospitalizations per 100,000 as smoke $PM_{2.5}$ concentration increases for blood diseases

© 2025 Vega SL et al. *JAMA Network Open*.

(A) Main model. (B) Main model with x-axis truncated at 30$\mu g/m^3$. (C) Main model fit on California. (D) Spline model fit on the full range of smoke $PM_{2.5}$ values with knots at 10, 25, and 40$\mu g/m^3$ and x-axis truncated at 50$\mu g/m^3$. (E) Spline model fit on the full range of smoke $PM_{2.5}$ values with knots at 10, 25, and 40$\mu g/m^3$. (F) Spline model with knots at 5, 10, 25$\mu g/m^3$. (G) Spline model with knots at 5, 20, 35$\mu g/m^3$. (H) Spline model with knots at 5, 10, 25, 40$\mu g/m^3$. (I) Main spline model with study period from May to December. (J) Poisson GLM model. (K) Binned model. (L) Binned model fit on full range of smoke $PM_{2.5}$ values.

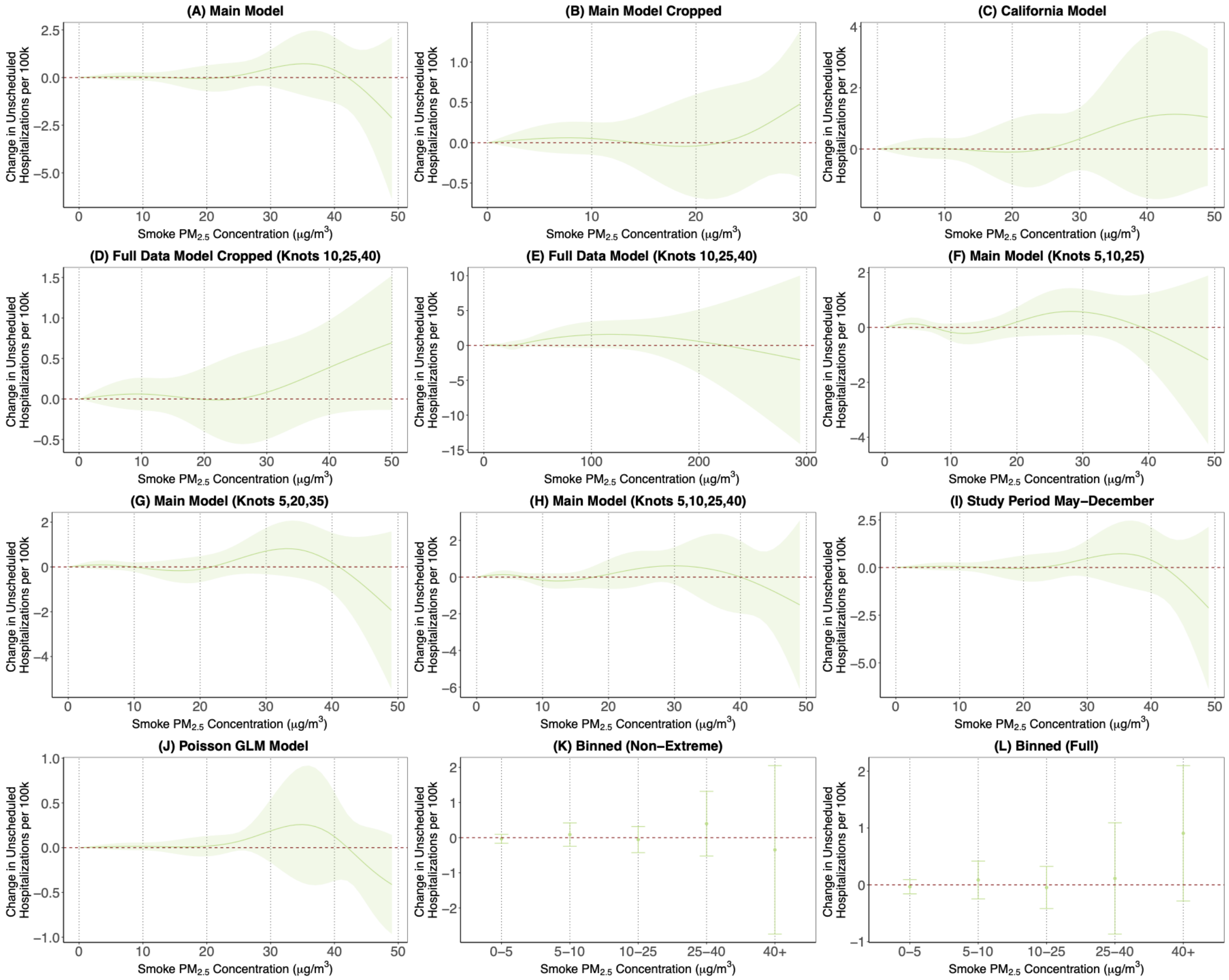

Figure S7: Changes in daily unscheduled hospitalizations per 100,000 as smoke $PM_{2.5}$ concentration increases for digestive system diseases

(A) Main model. (B) Main model with x-axis truncated at 30μg/m^3. (C) Main model fit on California. (D) Spline model fit on the full range of smoke $PM_{2.5}$ values with knots at 10, 25, and 40μg/m^3 and x-axis truncated at 50μg/m^3. (E) Spline model fit on the full range of smoke $PM_{2.5}$ values with knots at 10, 25, and 40μg/m^3. (F) Spline model with knots at 5, 10, 25μg/m^3. (G) Spline model with knots at 5, 20, 35μg/m^3. (H) Spline model with knots at 5, 10, 25, 40μg/m^3. (I) Main spline model with study period from May to December. (J) Poisson GLM model. (K) Binned model. (L) Binned model fit on full range of smoke $PM_{2.5}$ values.

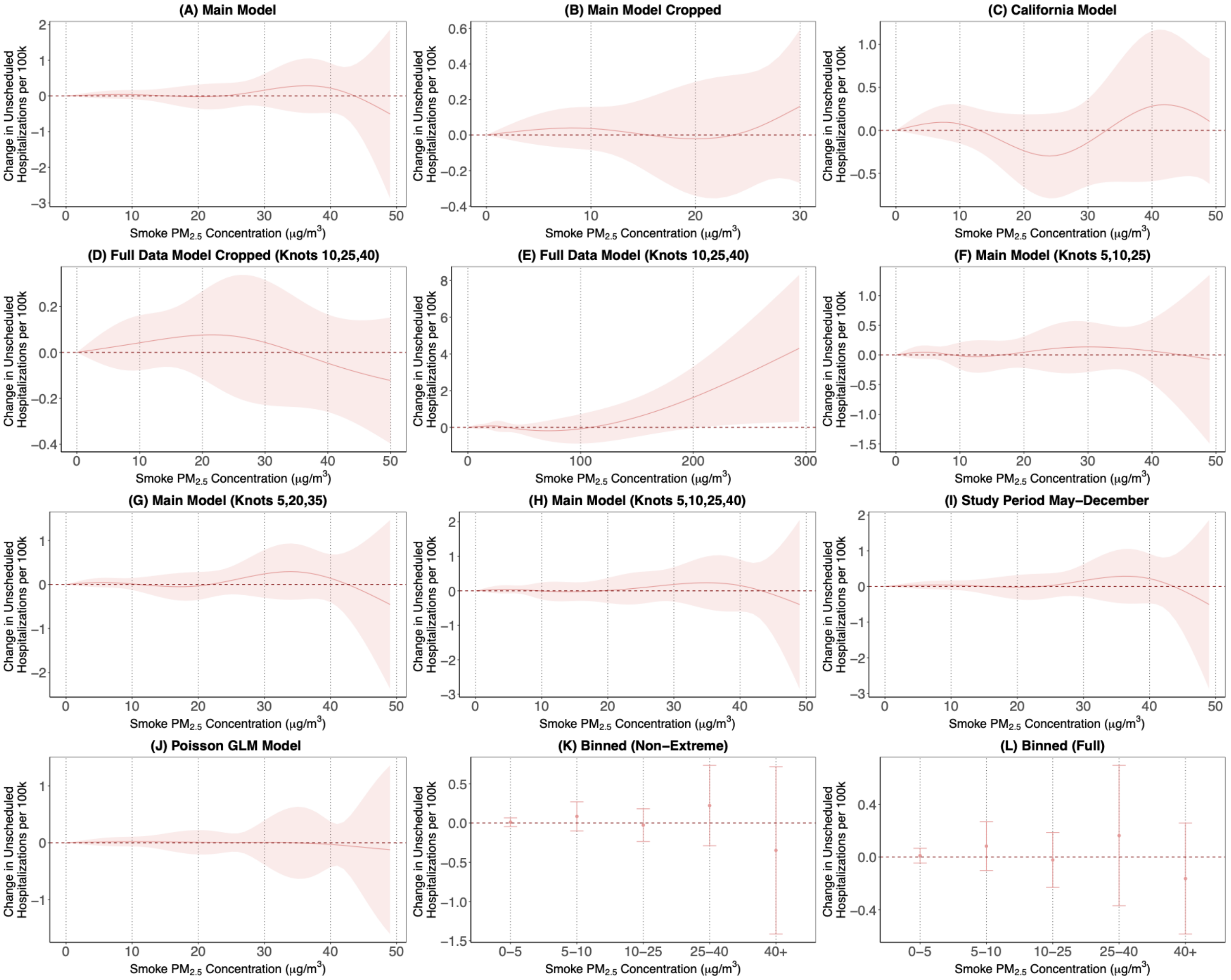

eFigure 8. Changes in daily unscheduled hospitalizations per 100,000 as smoke $PM_{2.5}$ concentration increases for endocrine disorders

(A) Main model. (B) Main model with x-axis truncated at 30μg/m^3. (C) Main model fit on California. (D) Spline model fit on the full range of smoke $PM_{2.5}$ values with knots at 10, 25, and 40μg/m^3 and x-axis truncated at

© 2025 Vega SL et al. *JAMA Network Open*.

50μg/m^3. (E) Spline model fit on the full range of smoke $PM_{2.5}$ values with knots at 10, 25, and 40μg/m^3. (F) Spline model with knots at 5, 10, 25μg/m^3. (G) Spline model with knots at 5, 20, 35μg/m^3. (H) Spline model with knots at 5, 10, 25, 40μg/m^3. (I) Main spline model with study period from May to December. (J) Poisson GLM model. (K) Binned model. (L) Binned model fit on full range of smoke $PM_{2.5}$ values.

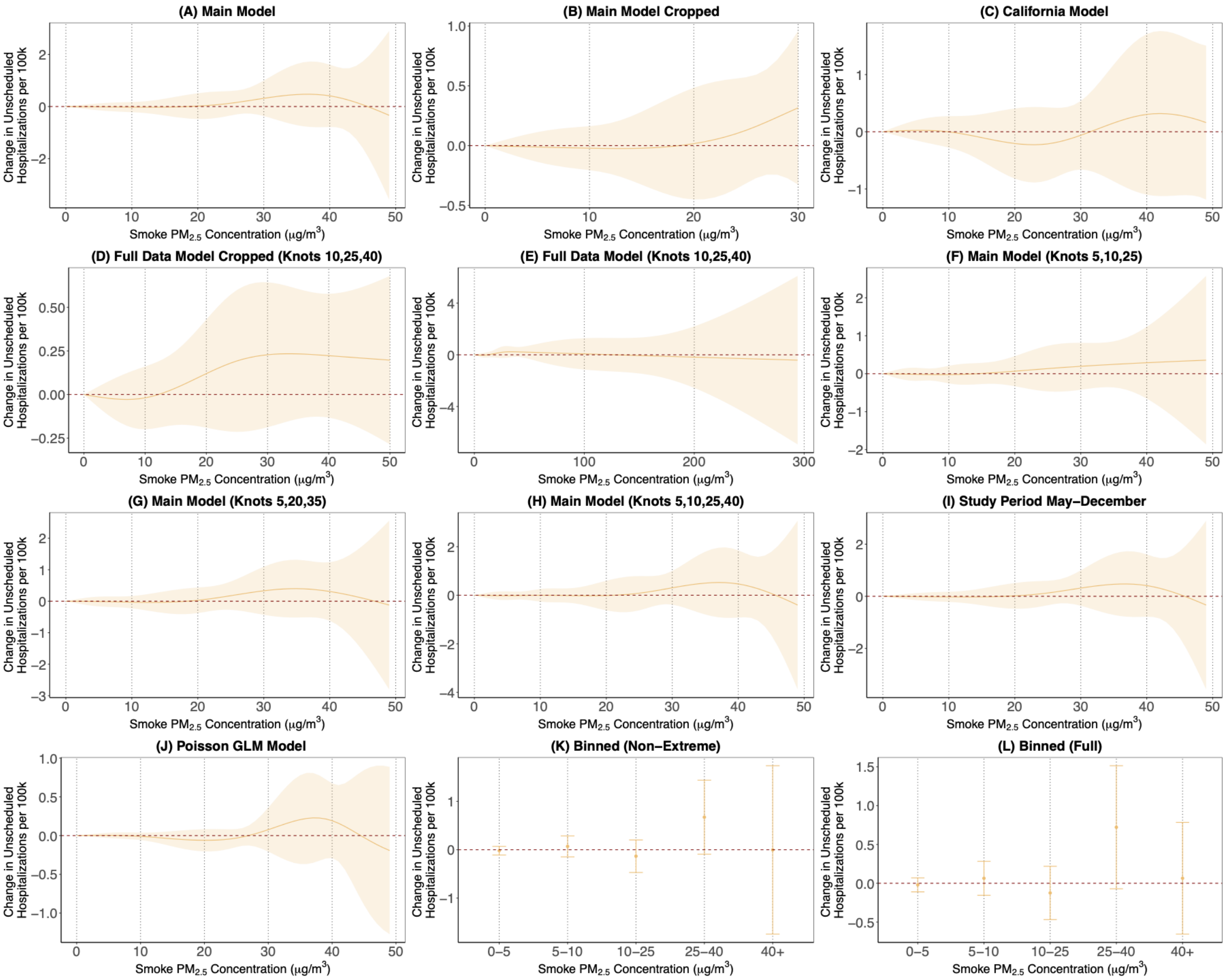

eFigure 9. Changes in unscheduled hospitalizations per 100,000 as smoke $PM_{2.5}$ concentration increases for genitourinary diseases

(A) Main model. (B) Main model with x-axis truncated at 30μg/m^3. (C) Main model fit on California. (D) Spline model fit on the full range of smoke $PM_{2.5}$ values with knots at 10, 25, and 40μg/m^3 and x-axis truncated at 50μg/m^3. (E) Spline model fit on the full range of smoke $PM_{2.5}$ values with knots at 10, 25, and 40μg/m^3. (F)

Spline model with knots at 5, 10, 25$\mu g/m^3$. (G) Spline model with knots at 5, 20, 35$\mu g/m^3$. (H) Spline model with knots at 5, 10, 25, 40$\mu g/m^3$. (I) Main spline model with study period from May to December. (J) Poisson GLM model. (K) Binned model. (L) Binned model fit on full range of smoke $PM_{2.5}$ values.

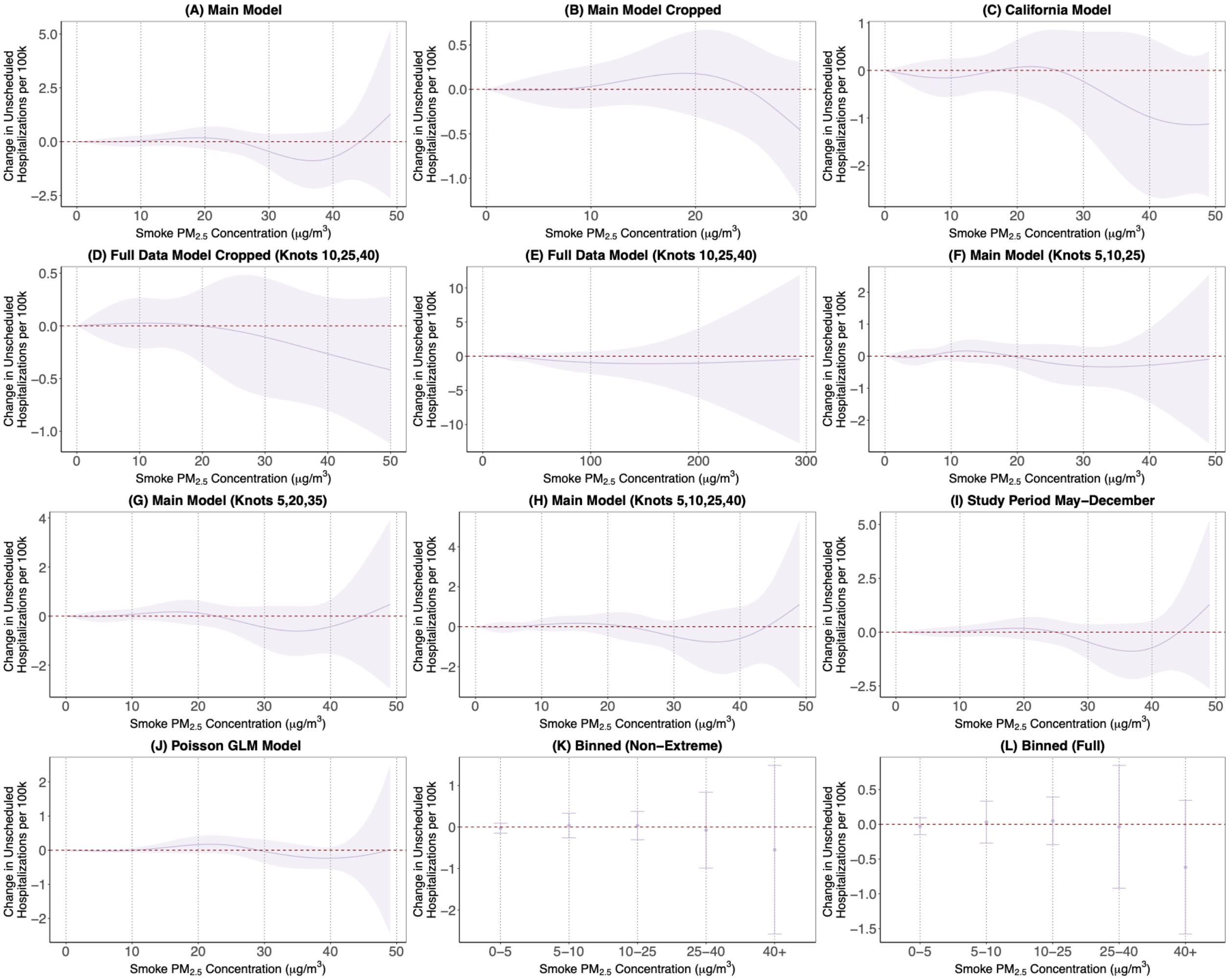

eFigure 10. Changes in daily unscheduled hospitalizations per 100,000 as smoke $PM_{2.5}$ concentration increases for infectious and parasitic diseases

(A) Main model. (B) Main model with x-axis truncated at 30$\mu g/m^3$. (C) Main model fit on California. (D) Spline model fit on the full range of smoke $PM_{2.5}$ values with knots at 10, 25, and 40$\mu g/m^3$ and x-axis truncated at 50$\mu g/m^3$. (E) Spline model fit on the full range of smoke $PM_{2.5}$ values with knots at 10, 25, and 40$\mu g/m^3$. (F) Spline model with knots at 5, 10, 25$\mu g/m^3$. (G) Spline model with knots at 5, 20, 35$\mu g/m^3$. (H) Spline model

© 2025 Vega SL et al. *JAMA Network Open*.

with knots at 5, 10, 25, 40μg/m³. (I) Main spline model with study period from May to December. (J) Poisson GLM model. (K) Binned model. (L) Binned model fit on full range of smoke $PM_{2.5}$ values.

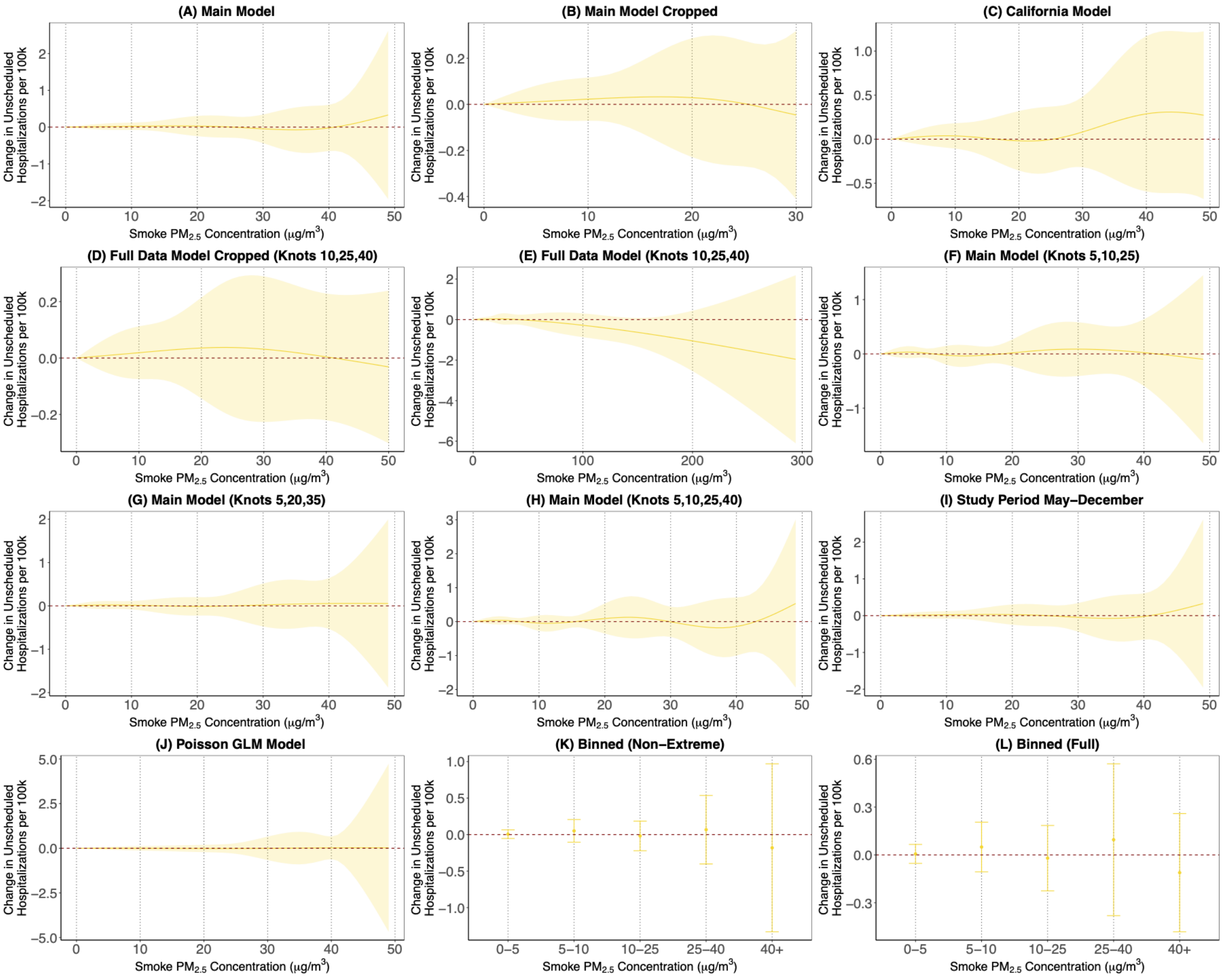

eFigure 11. Changes in daily unscheduled hospitalizations per 100,000 as smoke $PM_{2.5}$ concentration increases for musculoskeletal and connective tissue diseases.

(A) Main model. (B) Main model with x-axis truncated at 30μg/m³. (C) Main model fit on California. (D) Spline model fit on the full range of smoke $PM_{2.5}$ values with knots at 10, 25, and 40μg/m³ and x-axis truncated at 50μg/m³. (E) Spline model fit on the full range of smoke $PM_{2.5}$ values with knots at 10, 25, and 40μg/m³. (F) Spline model with knots at 5, 10, 25μg/m³. (G) Spline model with knots at 5, 20, 35μg/m³. (H) Spline model with knots at 5, 10, 25, 40μg/m³. (I) Main spline model with study period from May to December. (J) Poisson GLM model. (K) Binned model. (L) Binned model fit on full range of smoke $PM_{2.5}$ values.

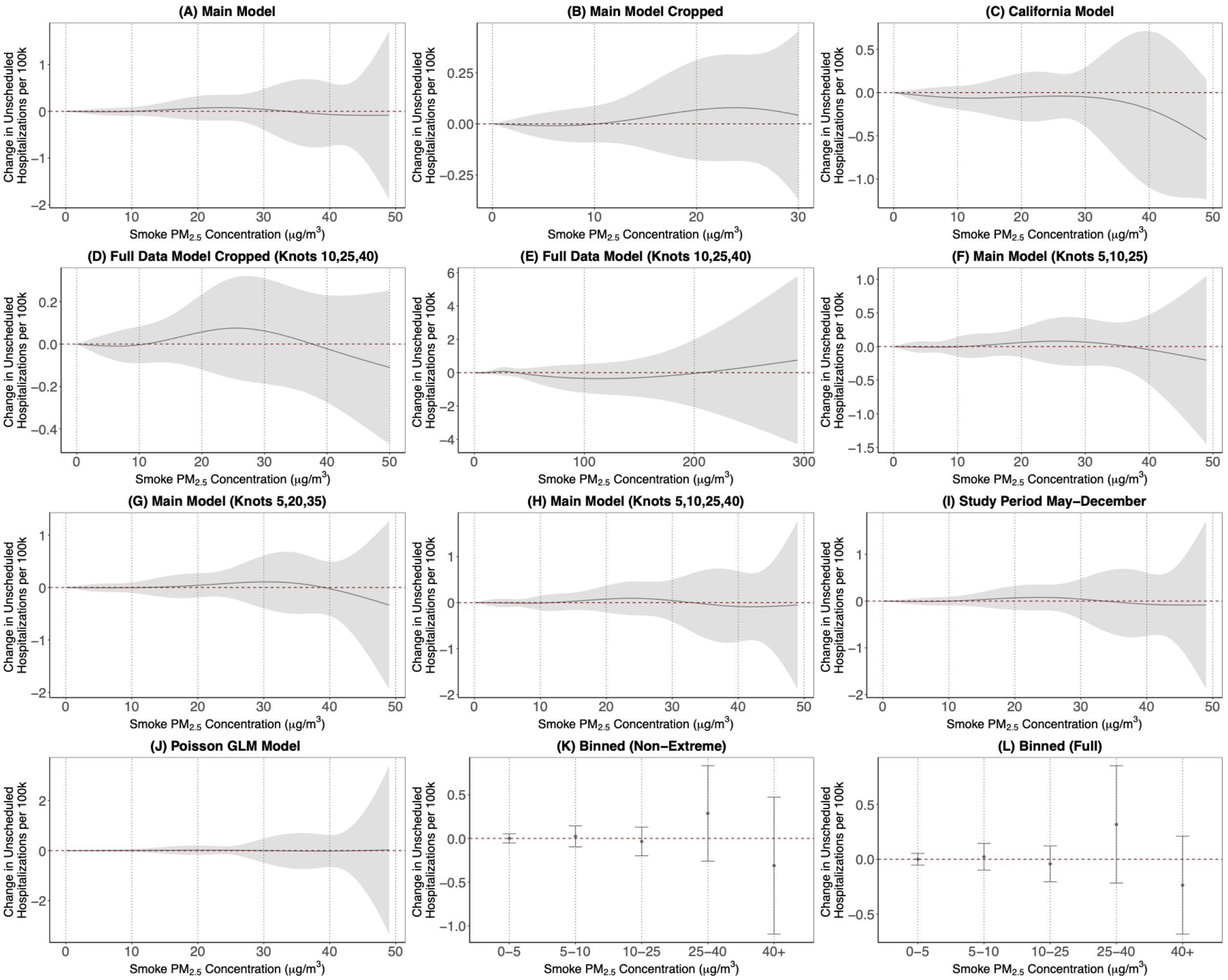

Figure S12: Changes in daily unscheduled hospitalizations per 100,000 as smoke $PM_{2.5}$ concentration increases for nervous system diseases

(A) Main model. (B) Main model with x-axis truncated at 30μg/m^3. (C) Main model fit on California. (D) Spline model fit on the full range of smoke $PM_{2.5}$ values with knots at 10, 25, and 40μg/m^3 and x-axis truncated at 50μg/m^3. (E) Spline model fit on the full range of smoke $PM_{2.5}$ values with knots at 10, 25, and 40μg/m^3. (F) Spline model with knots at 5, 10, 25μg/m^3. (G) Spline model with knots at 5, 20, 35μg/m^3. (H) Spline model with knots at 5, 10, 25, 40μg/m^3. (I) Main spline model with study period from May to December. (J) Poisson GLM model. (K) Binned model. (L) Binned model fit on full range of smoke $PM_{2.5}$ values.

© 2025 Vega SL et al. *JAMA Network Open*.

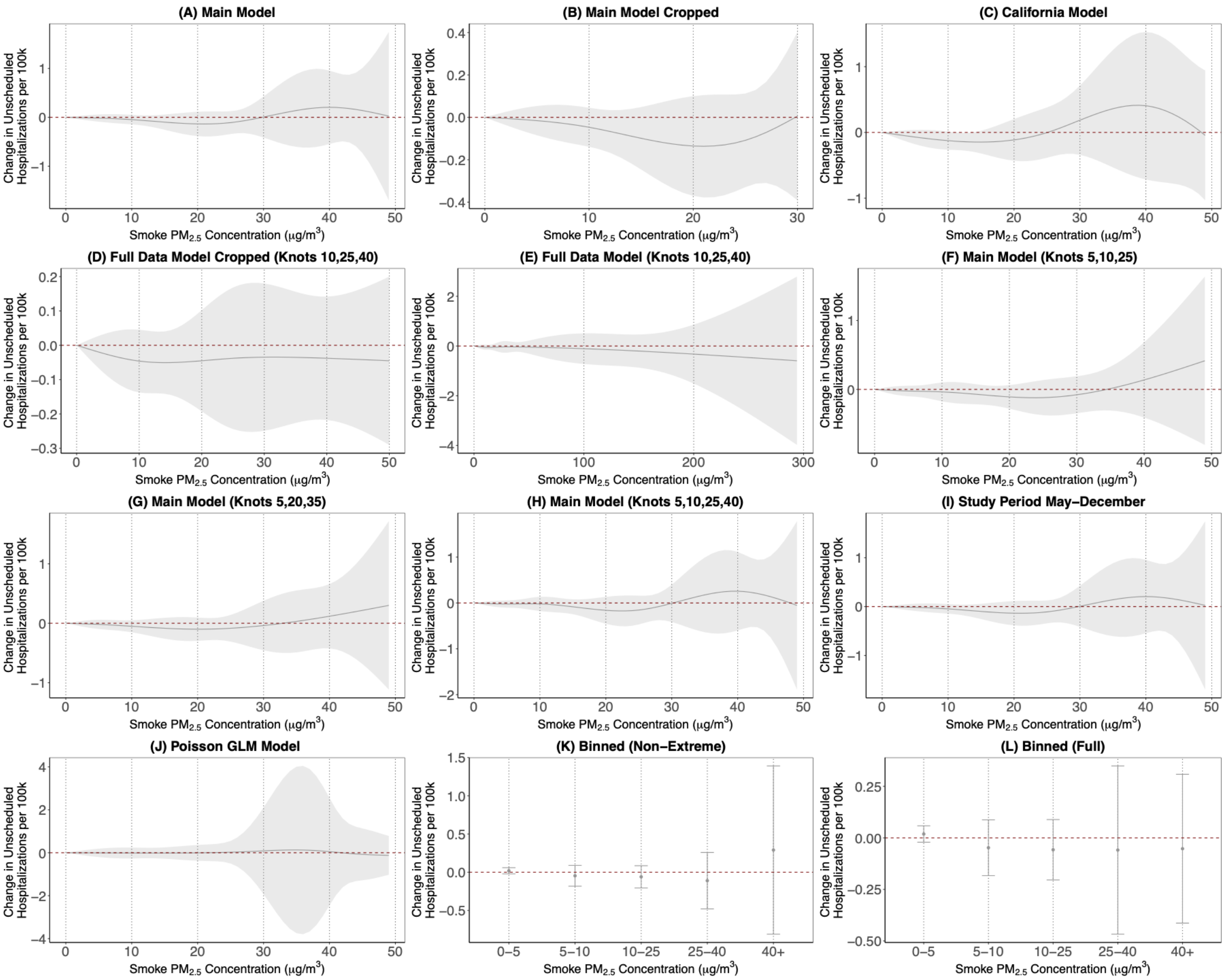

eFigure 13. Changes in daily unscheduled hospitalizations per 100,000 as smoke $PM_{2.5}$ concentration increases for skin and subcutaneous tissue diseases

(A) Main model. (B) Main model with x-axis truncated at $30\mu g/m^3$. (C) Main model fit on California. (D) Spline model fit on the full range of smoke $PM_{2.5}$ values with knots at 10, 25, and $40\mu g/m^3$ and x-axis truncated at $50\mu g/m^3$. (E) Spline model fit on the full range of smoke $PM_{2.5}$ values with knots at 10, 25, and $40\mu g/m^3$. (F) Spline model with knots at 5, 10, $25\mu g/m^3$. (G) Spline model with knots at 5, 20, $35\mu g/m^3$. (H) Spline model with knots at 5, 10, 25, $40\mu g/m^3$. (I) Main spline model with study period from May to December. (J) Poisson GLM model. (K) Binned model. (L) Binned model fit on full range of smoke $PM_{2.5}$ values.

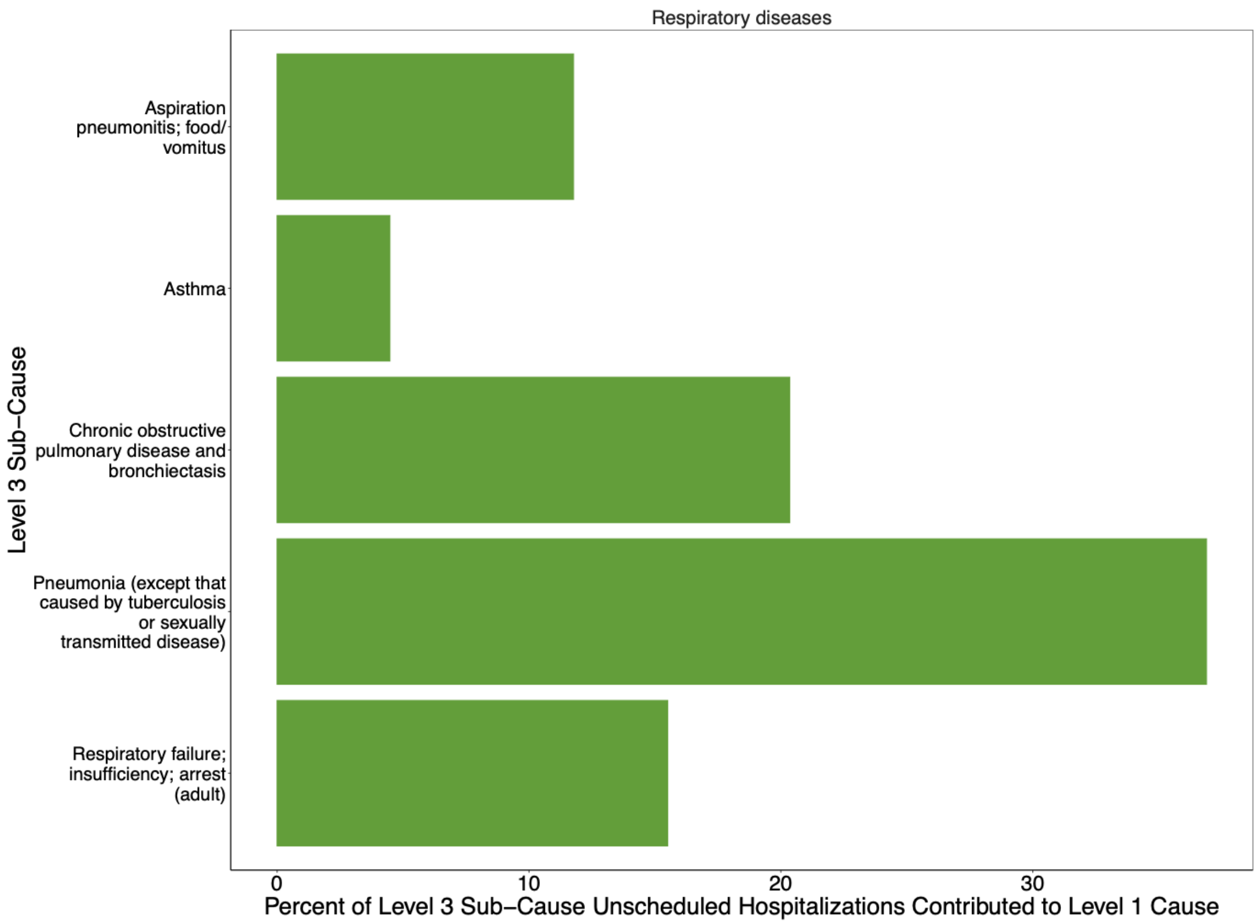

eFigure 14. Percentage of total unscheduled hospitalizations each sub-cause contributes to the respiratory disease broad cause.

© 2025 Vega SL et al. *JAMA Network Open*.

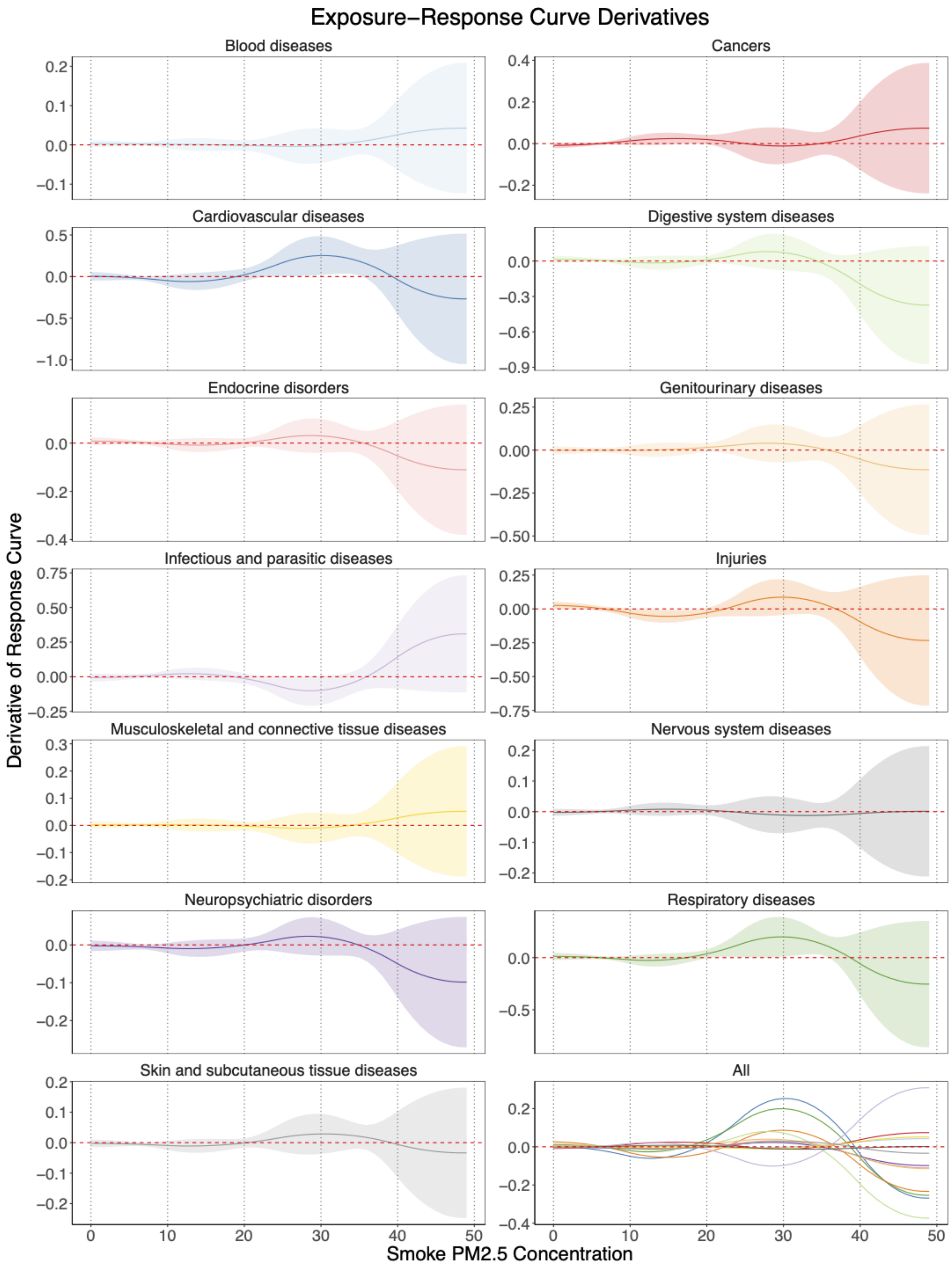
Exposure−Response Curve Derivatives
Blood diseases
Cancers
Cardiovascular diseases
Digestive system diseases
Endocrine disorders
Genitourinary diseases
Infectious and parasitic diseases
Injuries
Musculoskeletal and connective tissue diseases
Nervous system diseases
Neuropsychiatric disorders
Respiratory diseases
Skin and subcutaneous tissue diseases
All
Derivative of Response Curve
Smoke PM2.5 Concentration

eFigure 15. Point-wise derivatives of the estimated concentration-response curves for each outcome, and their 95% CIs

© 2025 Vega SL et al. *JAMA Network Open*.

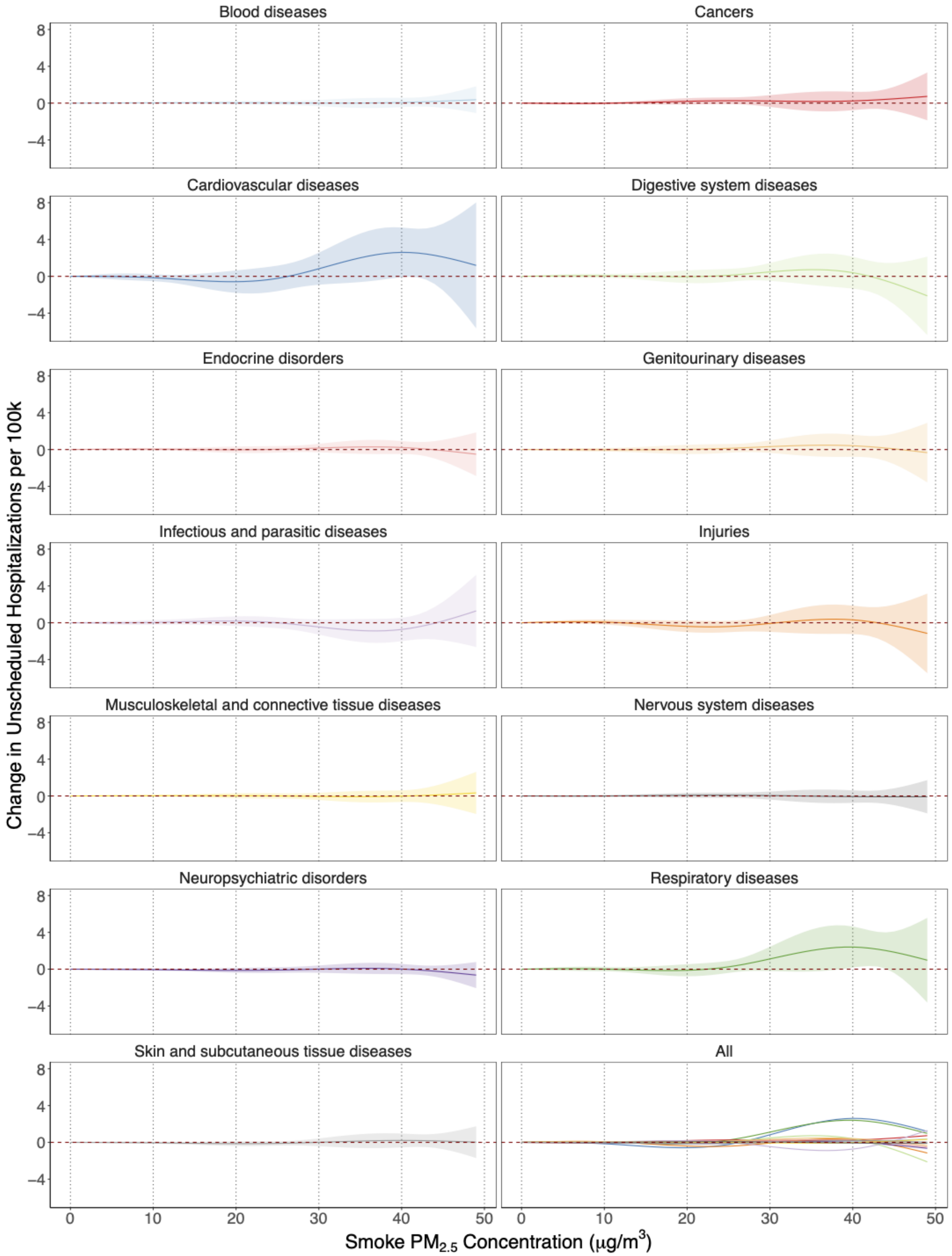

eFigure 16. Main model results plotted on the same y-axis

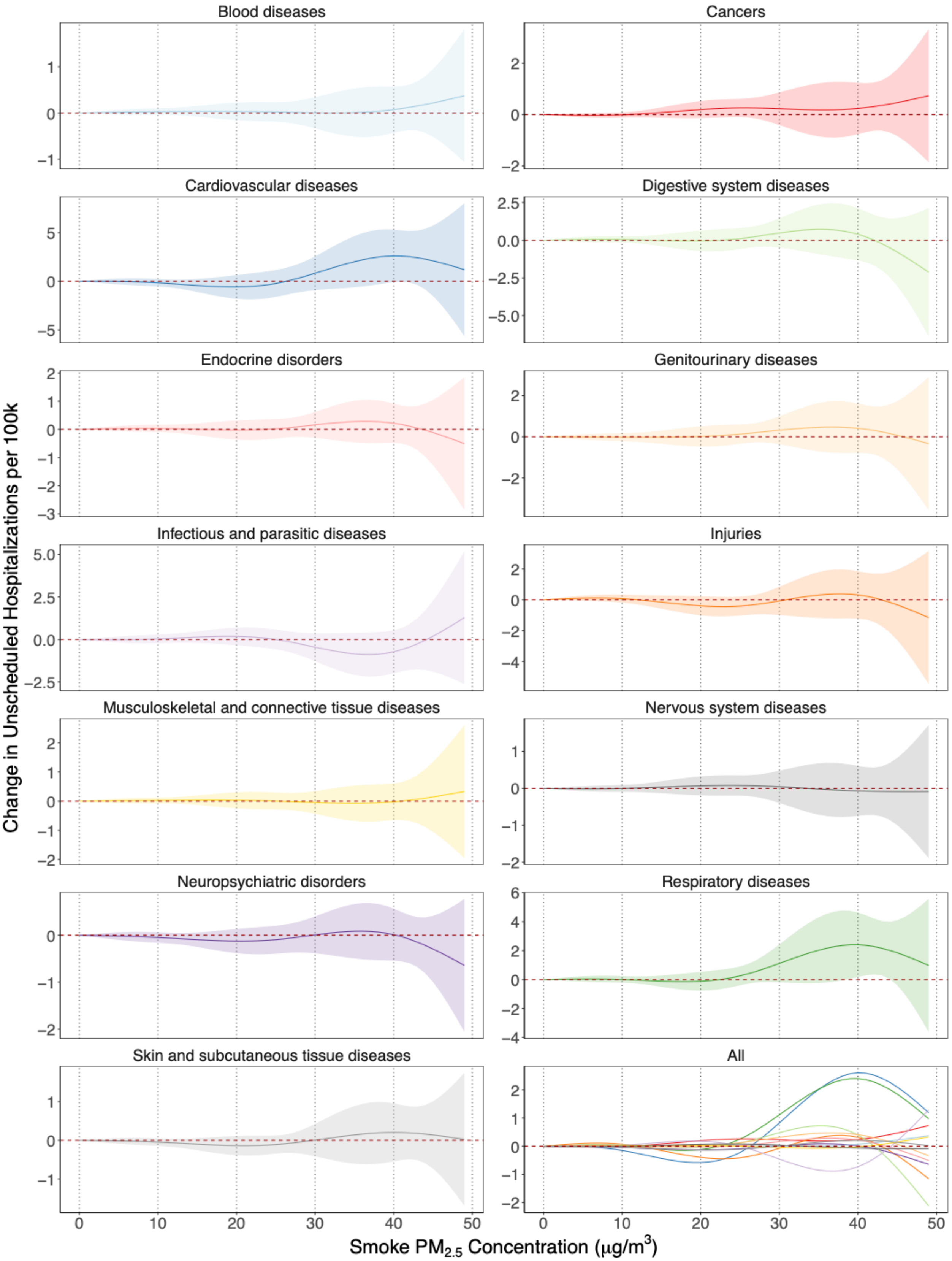

© 2025 Vega SL et al. *JAMA Network Open*.

eFigure 17. Estimated changes in daily unscheduled hospitalizations (per 100,000) for 13 causes associated with a given smoke $PM_{2.5}$ concentration, relative to 0, experienced on the same day and each of the preceding 7 days

Lines are point estimates and shaded areas are 95% confidence intervals, constructed using cluster robust standard errors.

eTable 1. Average daily rate of hospitalization (per 100,000 population) for each cause among the study population and time-period.

CAUSE	RATE
Blood diseases	0.42
Cancers	1.03
Cardiovascular diseases	7.92
Digestive system diseases	3.62
Endocrine disorders	1.24
Genitourinary diseases	2.08
Infectious and parasitic diseases	2.51
Injuries	3.52
Musculoskeletal and connective tissue diseases	0.73
Nervous system diseases	0.77
Neuropsychiatric disorders	0.56
Respiratory diseases	3.53
Skin and subcutaneous tissue diseases	0.57

eTable 2. Summary demographic information.

METRIC	VALUE
Male Percentage	45.99%
Female Percentage	54.01%
Mean Age	74.67
Dual Eligible Percentage	17.71%
Race - White Percentage	81.72%
Race - Black Percentage	3.25%
Race - Asian Percentage	5.87%
Race - Hispanic Percentage	4.02%
Race - North American Native Percentage	1.08%
Race - Other Percentage	3.25%

Race - Unknown Percentage	0.82%

eTable 3. The p-values from tests of residual auto-correlation for the primary model for each cause of hospitalization, where a p-value<0.05 indicates significant residual auto-correlation..

CAUSE	P-VALUE
Blood diseases	0.20
Cancers	0.01
Cardiovascular diseases	0.72
Digestive system diseases	0.74
Endocrine disorders	0.55
Genitourinary diseases	0.19
Infectious and parasitic diseases	0.06
Injuries	0.04
Musculoskeletal and connective tissue diseases	0.51
Nervous system diseases	0.59
Neuropsychiatric disorders	0.75
Respiratory diseases	0.17
Skin and subcutaneous tissue diseases	0.91

eTable 4. On-average change in rates of hospitalization for each cause (on both absolute and percent change scales) per 10 μg/m3 increase in smoke $PM_{2.5}$ across the exposure range computed empirically from the non-linear curve estimates in Figure 3.

CAUSE	Absolute Change in Rates	Percent Change
Blood diseases	0.03	7.14%
Cancers	0.13	12.62%
Cardiovascular diseases	0.54	6.82%
Digestive system diseases	0.18	4.97%
Endocrine disorders	0.08	6.45%
Genitourinary diseases	0.14	6.73%
Infectious and parasitic diseases	-0.20	-7.97%
Injuries	-0.02	-0.57%
Musculoskeletal and connective tissue diseases	-0.00	0.00%
Nervous system diseases	0.01	1.30%
Neuropsychiatric disorders	-0.03	-5.36%
Respiratory diseases	0.68	19.26%
Skin and subcutaneous tissue diseases	0.01	1.75%

© 2025 Vega SL et al. *JAMA Network Open*.
