# Supplementary material for: Wildfire Smoke Exposure and Cause-Specific Hospitalization in Older Adults
Source: JAMA Netw Open. 2025 Apr 30;8(4):e257956. doi: 10.1001/jamanetworkopen.2025.7956 (PMC12044514; doi:10.1001/jamanetworkopen.2025.7956)
Supplement: Supplement 2. — Data Sharing Statement [file jamanetwopen-e257956-s002.pdf]

Data Sharing Statement

Vega. Wildfire Smoke Exposure and Cause-Specific Hospitalization in Older Adults. *JAMA Netw Open*. Published April 30, 2025. doi:10.1001/jamanetworkopen.2025.7956

Data
Data available: No

Additional Information
Explanation for why data not available: Medicare enrollee data can be publicly accessed after purchasing and completing an application process through CMS. Smoke PM2.5 data is publicly available at [12]. Code to reproduce results is available at https://github.com/NSAPH-Projects/wildfires-hospitalizations-glm.
